# Diet and trophic structure of fishes in the Barents Sea: between empty and full stomachs – large individual variability follows a common pattern

**DOI:** 10.1111/jfb.16058

**Published:** 2025-01-17

**Authors:** Hein Rune Skjoldal, Elena Eriksen, Kotaro Ono, Andrey Dolgov

**Affiliations:** ^1^ Ecosystem Processes Research Group Institute of Marine Research Bergen Norway; ^2^ Fisheries Dynamics Research Group Institute of Marine Research Bergen Norway; ^3^ Polar branch of the Russian Federal Research Institute of Fisheries and Oceanography (“PINRO” named after N.M. Knipovich) Murmansk Russia

**Keywords:** fish feeding, fish size, individual variability, right‐skewed distribution, stomach content

## Abstract

More than 27,000 stomachs from 70 species of fish were collected from the Barents Sea in 2015. Quantitative stomach content expressed relative to the body weight of the predator fish (g g^−1^ as %) varied by four to five orders of magnitude for six species with the largest sample size (Atlantic cod *Gadus morhua*, haddock *Melanogrammus aeglefinus*, Greenland halibut *Reinhardtius hippoglossoides*, long rough dab *Hippoglossoides platessoides*, polar cod *Boreogadus saida*, and Atlantic capelin *Mallotus villosus*). The quantitative stomach contents of individual fish followed a common and strict statistical relationship for predator species or groups of species (by families), and for prey categories across predator species. The common pattern was log‐normal‐like and was modelled with good fit by different types of right‐skewed distributions, that is, variants of the Box–Cox, generalized inverse Gaussian, inverse gamma, or gamma distributions. The long tail in the high end reflects high variation with no clear sign of a plateau, as could be expected from the concept of a “full stomach”. This is interpreted to reflect that high stomach contents are rare events that are sampled at low frequencies. The maximum recorded stomach content varied from 1% to 34% of body weight for 55 species of fish, being positively correlated (*R* ^2^ = 0.45) with sample size. About a third of the stomachs were empty, and the low tail of the log‐normal‐like distribution represents the transition to empty stomachs. The amount of food in the stomachs was overall low compared to maximum values, with mean and median of 2.0% and 1.1%, respectively, for the 17,873 stomachs containing food. Supported by bioenergetic considerations, this suggests relatively low feeding rates of the various fish predators but sufficient to meet their energy demands.

## 1 INTRODUCTION

Stomach contents of fish provide information on what they have been eating in their environment. Analysis of stomach contents is a powerful way to examine trophic relationships and how species are connected in food webs (e.g., Buchheister & Latour, 2015; French et al., 2013; Garrison & Link, 2000). There is a long history of trophic investigations in the Barents Sea using diet analyses of fish stomachs to improve the basis for fisheries management through emphasis on multispecies interactions and the dynamics of the wider ecosystem (Dolgov, 2009, 2016; Jakobsen & Ozhigin, 2011; WGIBAR, 2021).

A comprehensive Norwegian–Russian data set on fish diet was collected in 2015 (dubbed “Year of the stomach”) through sampling of 27,627 individual stomachs from 70 fish species (Eriksen et al., 2020). The sampling consisted of two components: (1) the annual routine sampling of stomachs of commercially important species such as Atlantic cod (*Gadus morhua* L. 1758) and haddock (*Melanogrammus aeglefinus* [L. 1758]), and (2) supplementary sampling of other species, including non‐commercial species such as sculpins and eelpouts. We have in previous papers described general patterns of diet from stomach contents across the species sampled from the fish community of the Barents Sea (Eriksen et al., 2020, 2021, 2024). More than 200 species of fish are recorded from this area, but only ~100 of them are common and occur regularly (e.g., not as rare stray vagrants such as the ocean sunfish, *Mola mola* L. 1758) (Dolgov, 2016; Mecklenburg et al., 2018; Wienerroither et al., 2011). Thus, the sampling in 2015 included most of the common fishes of the Barents Sea.

Our previous studies have revealed a trophic structure of the Barents Sea fishes with clusters or guilds of planktivores, benthivores, and piscivores based on diet from the 2015 stomach sampling (Eriksen et al., 2020). The trophic structure has been found to be consistent and robust, with relatively small changes between geographical areas (e.g., Atlantic and Arctic domains) and seasons (Eriksen et al., 2021, 2024). Likewise, the trophic structure remained largely unchanged when individual size was included, with predator groups being combinations of species and size groups compared to species only without size information (Eriksen et al., 2024). The general patterns of trophic structure were extracted from data where individual variability had been removed or reduced by averaging across individuals within species or species and length groups. The diet was also expressed in relative terms as weight percentages of prey components of the total stomach content.

In the present study, the focus is on the individual variability in the amount of food in fish stomachs for different types of prey (e.g., plankton, benthos, fish) and across different species and trophic types of fish predators. The individual variability reflects the complexity of fish feeding in the diverse setting of their ecological environment and has implications for the interpretation and statistical treatment of stomach content data. We address the following main questions: (1) Is there an underlying statistical pattern (probability density function) for the individual variation in the amount of food in fish stomachs? (2) If so, what is this statistical pattern? (3) Does the pattern vary for different types of prey and different species, taxonomic groups, and trophic types of fish? (4) How is the individual variation in stomach content affected by the size of the individual fish predators? By addressing these questions, the main objective was to characterize and quantify the individual variability in the weight of stomach content in relation to the species and size of the fish predators and different types of prey (plankton, benthos, and fish). In the discussion, we emphasize the two extreme ends of the distributions: empty stomachs and full stomachs.

## 2 MATERIALS AND METHODS

### 2.1 Sampling and stomach analysis

Fish were collected with bottom or pelagic trawls in the Barents Sea. Most fish were collected during two joint Norwegian–Russian monitoring surveys, one in winter (January–March) and the other (an ecosystem survey) in autumn (August–October) (Eriksen et al., 2018). Sampling was also conducted on scientific cruises in spring and summer and supplemented with samples from Russian commercial fisheries (see Eriksen et al., 2020 for more details).

The fish were sorted from the trawl samples and processed as quickly as possible after retrieval of the trawl on deck. Sampling of stomachs from commercial species followed a length‐stratified procedure to include all length groups in samples. The additional sampling of non‐commercial species (or species of low commercial importance and not included in the routine stomach sampling) was done in an ad hoc and opportunistic manner, trying to sample as many species as possible depending on the availability of species in samples and capacity (time and personnel) to process them. If available, 10 specimens of different sizes were selected for each additional species from a trawl sample. Fish larger than 12 cm in length were processed directly with the fresh samples, while smaller individuals (<12 cm) were frozen and stored for later analysis in the laboratory ashore.

The contents of individual stomachs were weighed and further analyzed by identifying and sorting prey to the highest possible taxonomic resolution, depending on the degree of digestion and the skill of the personnel analyzing the samples. The sorted prey groups were weighed separately.

### 2.2 The 2015 fish diet data set

A total of 70 species of fish, including two genera, were sampled for a total number of 27,627 stomachs. The sample size was very uneven across species, ranging from only one individual for seven of the species to a maximum of 11,557 stomachs for Atlantic cod. A list of the fish species with information on sample size is provided in Eriksen et al. (2020), their Table 2. The number of stomachs containing food was 17,876 for a total of 67 species; 9751 (35%) stomachs were empty.

The taxonomic resolution of identified prey in the stomach contents ranged from species to phylum, with more than 350 prey types recognized across the whole sample material (a list of these prey types is found as supplementary material in Eriksen et al., 2020). We grouped the prey types into 12 categories at higher taxonomic levels from order to phylum (Table 1). Five of the prey categories were zooplankton and another five were predominantly benthic animals. All fish prey were grouped into one category. In addition, we recorded digested food that could not be recognized to any of the 12 identified prey categories.

**TABLE 1:** Twelve prey categories were used to describe the diet composition of fish predators in the 2015 stomach sampling study.

Prey category	Taxonomic level	Dominant species or groups
Copepods	Class	*Calanus finmarchicus*, *C. glacialis*
Euphausiids	Order	*Meganyctiphanes norvegica*, *Thysanoessa inermis*, *T. raschii*
Hyperiids	Suborder (Order Amphipoda)	*Themisto abyssorum*, *T. libellula*
Gelatinous zooplankton	Phylum	Ctenophores
Other zooplankton	Phylum	Chaetognaths, pteropods
Small demersal crustaceans	Phylum/sub‐phylum	Gammarid amphipods, isopods
Large demersal crustaceans	Phylum/sub‐phylum	Shrimps (*Pandalus borealis*, and others) and crabs
Worms	Phylum/class	Polychaetes
Echinoderms	Phylum	Brittle stars, sea cucumbers
Molluscs	Phylum	Bivalves, gastropods
Fish	Sub‐phylum	Capelin, polar cod, Atlantic cod, long rough dab, daubed shanny, and others
Other prey	Phylum	Cephalopods, other prey, including unidentified items
Digested food		Strongly digested and not identifiable prey

*Note*: The prey categories were aggregated at a high taxonomic level based on raw data with higher (but variable) taxonomic resolution (see Eriksen et al., 2020).

The data set used in this study is a matrix of 14 columns by 17,873 rows (three stomachs out of the 17,876 stomachs with food were excluded due to only digested food). The rows are the individual fish and stomachs analyzed for diet composition. The columns are the 12 identified prey categories plus digested food and total stomach contents, expressed as the fresh (wet) weight of each prey category standardized to the weight (wet) of the individual fish from which the stomach was removed. The unit is g/g expressed as the weight of a prey category or total stomach content as percentage of the wet weight of the individual fish.

### 2.3 Data exploration and analyses

We use the data set in two different ways:

1. The total data set with diet composition for 17,873 individual fish from 67 species (each with from one to 8969 stomachs).
2. Data separated for six dominant species with high numbers of analyzed stomachs (Atlantic cod, 8969 stomachs; haddock, 2527; capelin *Mallotus villosus* (Müller 1776), 1078; Greenland halibut *Reinhardtius hippoglossoides* (Walbaum 1792), 712; long rough dab *Hippoglossoides platessoides* (Fabricius 1780), 655; polar cod *Boreogadus saida* (Lepechin 1774), 609) or grouped by taxonomy (family) for species with fewer stomachs.

The six species differ in their habitat association and trophic ecology. Cod and haddock are demersal species, polar cod and Greenland halibut are benthopelagic, long rough dab is benthic, while capelin is a pelagic species. Cod and Greenland halibut tend to be piscivores, haddock and long rough dab benthivores, and polar cod and capelin planktivores (Eriksen et al., 2020, 2024).

The data for the weight of stomach contents of the 12 prey categories and for weight of total stomach content across all stomachs were examined with data both on a linear scale and after log‐transformation (log10). Statistical summaries were prepared with information on mean, median, and maximum stomach content for the various prey categories and total stomach content with linear‐scale data. Data were displayed as frequency histograms (linear scale) and as ranked plots of individual stomachs from highest to lowest recorded stomach content (log scale).

Summary statistics with data on linear scale were provided for the weight of total stomach content for all species (55), including mean and maximum stomach content. Data for the six selected species with high sample size were treated in a similar manner to the data on prey categories, with frequency histograms and ranked plots for total stomach content. Ranked plots (log scale) were also prepared for groups of species by fish families or groups of families (see legend to Figure 5). Additionally, more than 20 parametric distributions were fitted to the stomach content data (excluding empty stomachs) for the above six species using the function *fitDist()* in the R package *GAMLSS* (Rigby & Stasinopoulos, 2005), and the distribution with the lowest AIC was kept for examination. The same analysis was also done with the data for stomach content of the 12 prey categories across all fish predator species (stomachs) which contained each of the prey types.

Variation in the weight of stomach contents for prey categories and total stomach content in relation to size of the individual fish (body weight) was examined with regression analysis for each of the six selected species. Quantile regression analysis was used to describe trends for the median and upper (90%) and lower (10%) quantiles.

The variation in weight of total stomach content across the individuals of all species was examined using random forest analysis (Wright & Ziegler, 2017), which is a machine learning approach. The analysis examined how the factors individual size (body weight), species (*n* = 66), individual sampling station (*n* = 1401), season (*n* = 4), and geographical area (*n* = 15) (see Eriksen et al., 2024) contributed to explaining the variability in individual stomach content.

Descriptive statistics and some plots were done in Microsoft Excel. All other analyses were performed using R (R Core Team, 2024).

### 2.4 Ethics statement

The sampling of fish for the 2015 stomach content data set was carried out on governmental research vessels in full accordance with national and international guidelines and standards through the International Council for the Exploration of the Sea.

## 3 RESULTS

### 3.1 Quantitative stomach contents by prey groups: statistical summary for the total data set

**FIGURE 1:**
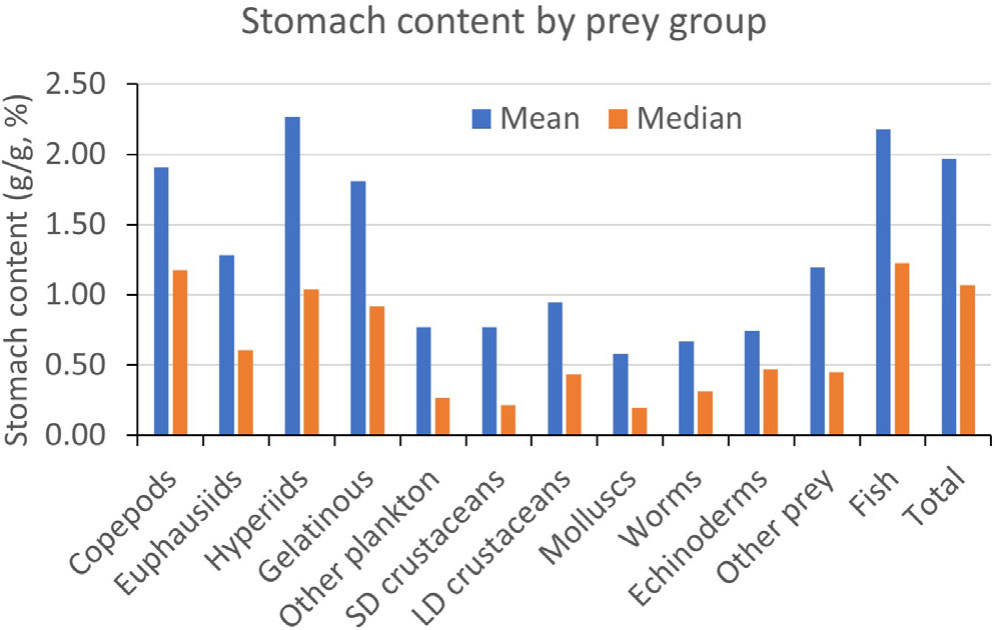
Weight of stomach contents (arithmetic mean and median; g prey per g fish weight, %) for 12 prey categories and the total stomach content. The mean and median values are calculated for the number of stomachs that contained each of the prey categories (see Table 2 for the number of stomachs). The total stomach content is for all stomachs containing food (*n* = 17,873).

Each of the 12 prey categories occurred in 5% (other prey) to 33% (fish) of the total number of stomachs (17,873) (Table 2). Each of the five prey categories of plankton and benthos, respectively, occurred in 8% to 15% of the stomachs. The arithmetic mean stomach contents of each prey category, for the number of stomachs that contained such prey, ranged from 0.6% (molluscs) to 2.3% (hyperiids) (Table 2 and Figure 1). The corresponding median values were lower than the means (typically ~half), varying from 0.2% (molluscs, small benthic crustaceans) to 1.2% (fish, copepods) (Table 2 and Figure 1). The mean and median values for total stomach content were 2.0% and 1.1%, respectively (Table 2). Stomach content tended to be higher for the groups of plankton prey (copepods, euphausiids, hyperiids, gelatinous plankton) compared to benthic prey (small demersal [SD] and large demersal [LD] crustaceans, molluscs, worms, and echinoderms), with mean values of ~1%–2% and ~0.5%–1.0%, respectively (Figure 1).

**TABLE 2:** Stomach contents by prey group across the total data set of 17,873 stomachs collected from 67 species of fish in the Barents Sea in 2015.

Prey group	Number of stomachs	% total	Mean	Median	Median/mean	SD	Maximum	Max 5	% <0.5	% >10
Copepods	1380	7.7	1.91	1.18	0.62	2.35	30.8	18.4	32.6	0.94
Euphausiids	2779	15.5	1.28	0.61	0.47	1.90	22.7	19.8	46.1	0.50
Hyperiids	1626	9.1	2.27	1.04	0.46	3.26	30.0	27.3	35.9	3.26
Gelatinous plankton	1475	8.3	1.81	0.92	0.51	2.39	18.1	16.3	34.6	1.97
Other plankton	1381	7.7	0.77	0.27	0.35	1.66	33.5	16.8	65.5	0.29
Fish	5911	33.1	2.18	1.23	0.56	2.62	23.4	20.6	28.1	2.05
Small demersal crustaceans	1370	7.7	0.77	0.22	0.28	1.60	32.1	14.9	66.0	0.15
Large demersal crustaceans	2592	14.5	0.94	0.43	0.46	1.50	19.1	16.0	54.1	0.39
Molluscs	1335	7.5	0.58	0.20	0.34	1.22	14.1	11.5	74.0	0.22
Worms	2380	13.3	0.70	0.31	0.45	2.08	29.6	14.8	64.9	0.17
Echinoderms	1784	10.0	0.74	0.47	0.63	0.93	8.3	8.0	52.1	0.00
Other prey	898	5.0	1.19	0.45	0.38	1.99	12.1	13.3	52.8	0.78
Digested food	588	3.3	0.82	0.37	0.45	1.32	15.1	9.1	60.5	0.17
Total	17,873	100	1.98	1.07	0.54	2.64	33.5	31.3	29.3	1.77

*Note*: Statistical summaries of the number of stomachs containing prey categories (numbers and % of total number of stomachs) and arithmetic mean, median, standard deviation, and maximum % weight (g stomach content/g fish weight, as percentage) of prey categories. Column “Max 5” gives the average stomach content of the five stomachs with the highest contents. The two last columns give the percentage of stomachs containing <0.5% and >10% stomach content weight, respectively. Note that the weights of stomach contents are given for the numbers of stomachs that contained each of the prey categories. Two outliers with exceptionally high stomach contents were removed for Liparis bathyarcticus and Lycodes rossi (66 % and 83 %, respectively).

### 3.2 Frequency distribution of stomach contents across individuals on a linear scale

**FIGURE 2:**
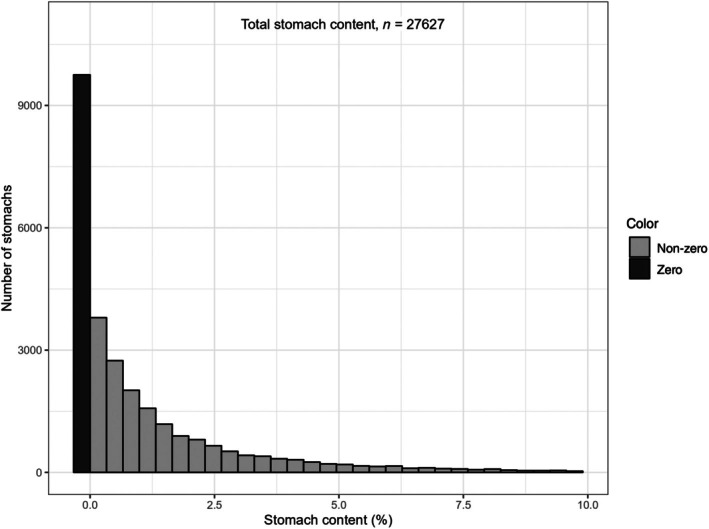
Frequency histogram of number of empty stomachs (*n* = 9751) and number of stomachs containing food (17,873) versus total weight of stomach content. The scale is cut at 10%, which excludes 1.8% of the stomachs with content >10%, out to a maximum stomach content of 33.5% (Table 2).

As indicated by low median/mean ratios (Table 2), the frequency distributions of stomach contents were highly right‐skewed with a long tail in the direction of high stomach content. For total stomach content across all 17,873 stomachs, the number of stomachs decreased in an exponential fashion with increasing stomach content (Figure 2). Twenty‐nine percent of the stomachs (with food) contained less than 0.5% food (on a weight‐basis) (Figure 2 and Table 2), and nearly 50% (48%) contained <1%. If we add the number of empty stomachs (9751), these percentages increase to 54% (<0.5% stomach content) and 66% (<1% stomach content).

The frequency distributions for the weight of stomach contents for each of the 12 prey categories showed similar patterns of exponential‐like decrease in number of stomachs versus increasing stomach contents (Figure S1). Between 28% (fish) and 74% (molluscs) of stomachs with food of each prey category had stomach content <0.5% of that prey category (Table 2).

### 3.3 Maximum stomach content of prey categories

The maximum content in individual stomachs for prey of the different categories ranged from 8% (echinoderms) to 34% (other plankton), with most values around 20%–30% (Table 2). With a large data set, there is a possibility that some of the highest values are erroneous due to errors in recorded weight of prey (too high) or predator fish (too low). To reduce the influence of any erroneously high values, we calculated the mean contents of the five highest values for the stomachs. These values ranged from 8% (echinoderms) to 27% (hyperiids) (Table 2).

Few stomachs had high content. Thus, the percentage of stomachs containing >10% food on weight basis varied from 0% (echinoderms) to 3.3% (hyperiids) for the different prey categories (Table 2). About 2% of the stomachs with fish prey had content exceeding 10%. Generally, more stomachs had high content for the plankton prey categories (0.3%–3.3% of stomachs with more than 10% content) than the benthic prey categories (0.0%–0.4%) (Table 2).

### 3.4 Mean and maximum stomach contents by species of fish predators

**FIGURE 3:**
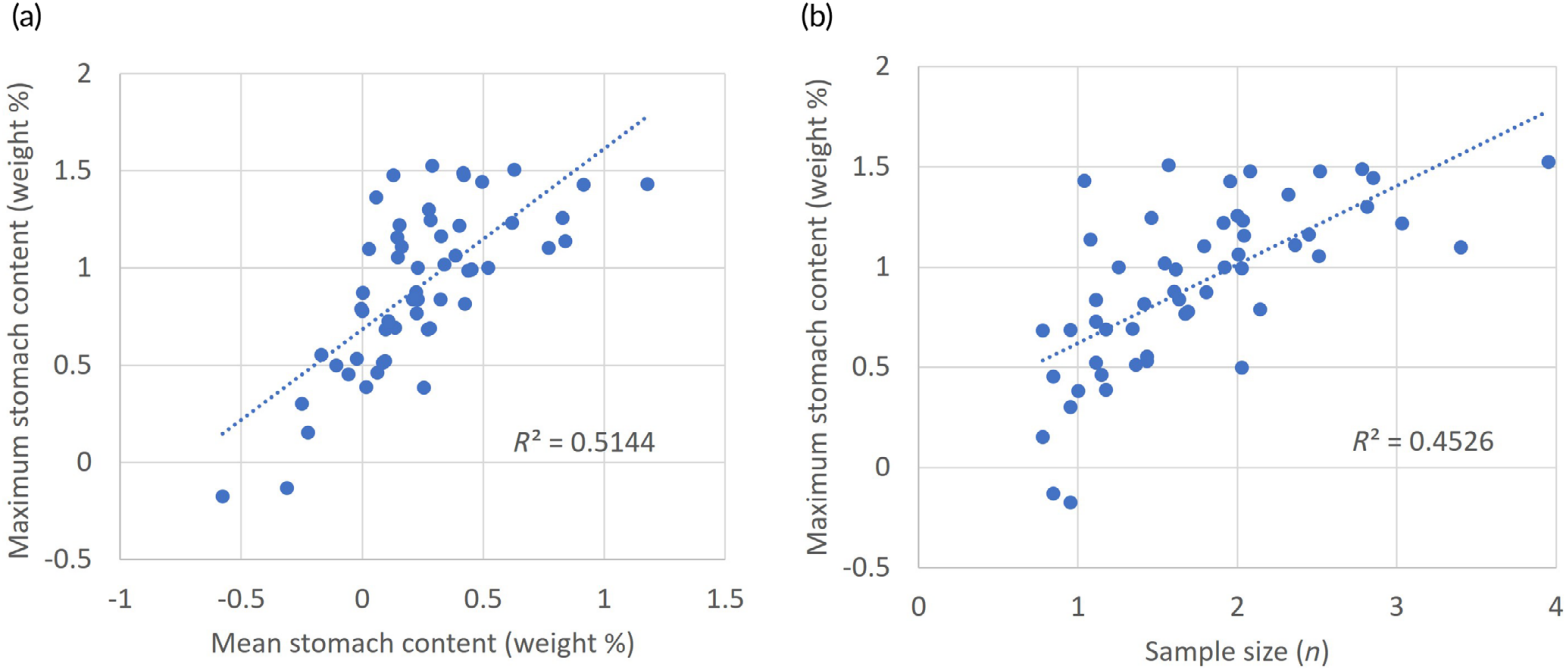
Scatter plot and linear regression for relationships between (a) maximum and mean stomach contents (weight‐%), and (b) maximum stomach content and sample size (*n* = number of stomachs) for average values for 55 species of fish predators with sample size *n* > 5 (Table 3). Values are log10 transformed.

The arithmetic mean stomach content for the 55 species of fish predators with sample size of more than five stomachs (*n* = 6–8969) varied from 0.3 to 15.1 weight‐%, with an overall mean of 2.3 (standard deviation 2.4) weight‐% (Table 3). The mean stomach content was strictly correlated with the associated standard deviation values across the 55 species (*R* ^2^ = 0.77, regression slope *b* = 0.96 with log‐transformed data). The maximum stomach content for the same 55 species varied from 0.7 to 33.5 weight‐%, with an overall mean of 11.6 (standard deviation 9.2) weight‐% (Table 3). The mean and maximum stomach contents for the 55 species were strongly correlated with *R* ^2^ = 0.51 for log‐transformed data (Figure 3a). The maximum stomach content was also significantly and positively correlated with sample size (*R* ^2^ = 0.45; Figure 3b), while the mean stomach content was not (*R* ^2^ = 0.03).

**TABLE 3:** Arithmetic mean, median, maximum, and standard deviation of total stomach content (% weight) for 55 species of fish predators with sample size (number of stomachs, *n*) >5.

Species	Mean	Median	Standard deviation	Maximum	*n*	Cluster
*Amblyraja hyperborea*	1.62	1.35	1.88	6.88	13	1
*Bathyraja spinicauda*	1.87	1.06	1.85	4.84	6	1
*Eutrigla gurnardus*	0.60	0.50	0.46	1.42	6	1
*Gadus morhua*	1.95	1.10	2.38	33.51	8969	1
*Liparis bathyarcticus*	6.92	6.31	3.36	13.75	12	1
*Lycodes reticulatus*	2.10	1.29	2.10	6.87	13	1
*Micromesistius poutassou*	2.11	1.46	2.19	14.57	282	1
*Osmerus dentex*	5.88	6.15	2.74	12.70	62	1
*Pollachius virens*	1.39	0.40	2.33	14.37	110	1
*Reinhardtius hippoglossoides*	3.13	2.15	3.24	27.78	712	1
*Sebastes mentella*	1.40	0.80	1.74	11.32	325	1
*Sebastes norvegicus*	1.80	1.88	0.66	2.42	10	1
*Ammodytes marinus*	3.31	2.79	2.06	10.00	18	2
*Clupea harengus harengus*	2.43	1.82	2.11	11.59	102	2
*Clupea pallasii suworowi*	1.36	0.92	1.31	4.93	22	2
*Gadiculus argenteus thori*	1.68	1.25	1.39	5.83	47	2
*Mallotus villosus*	2.52	1.60	2.67	16.50	1078	2
*Sebastes viviparus*	1.91	1.71	1.37	4.90	15	2
*Trisopterus esmarkii*	0.78	0.55	0.74	3.15	107	2
*Boreogadus saida*	2.62	1.54	3.04	30.77	609	3
*Eumicrotremus derjugini*	15.09	17.42	8.83	27.00	11	3
*Eumicrotremus spinosus*	8.20	8.59	4.83	26.82	90	3
*Liparis fabricii*	4.17	3.99	3.40	17.07	108	3
*Lycodes pallidus*	1.24	1.10	0.98	3.33	13	3
*Triglops murrayi*	1.70	1.11	2.05	10.00	83	3
*Triglops nybelini*	2.63	1.00	4.16	30.00	120	3
*Triglops pingelii*	1.70	1.33	1.48	6.91	43	3
*Anarhichas denticulatus*	1.91	0.81	3.53	17.59	29	4
*Arctozenus risso*	0.88	0.50	1.09	2.84	7	4
*Argentina silus*	1.01	0.67	1.28	7.48	64	4
*Cyclopterus lumpus*	6.74	6.28	4.26	18.08	100	4
*Artediellus atlanticus*	1.35	0.56	2.53	30.00	329	5
*Leptoclinus maculatus*	1.43	0.67	2.47	16.67	82	5
*Limanda limanda*	2.65	2.34	1.71	6.56	26	5
*Liopsetta glacialis*	1.04	0.76	0.60	2.44	15	5
*Lumpenus lampretaeformis*	1.00	0.68	1.02	6.00	49	5
*Lycodes rossi*	1.21	1.04	0.97	3.26	23	5
*Microstomus kitt*	0.49	0.64	0.24	0.74	7	5
*Rajella fyllae*	1.28	1.01	1.32	5.33	13	5
*Amblyraja radiata*	1.45	0.97	1.73	12.85	230	6
*Brosme brosme*	1.25	0.43	1.55	4.85	9	6
*Eleginus nawaga*	2.84	2.04	2.37	9.84	107	6
*Macrourus berglax*	0.26	0.22	0.25	0.67	9	6
*Aspidophoroides olrikii*	0.56	0.33	0.62	2.00	9	7
*Careproctus* spp.	4.25	2.73	5.47	32.14	37	7
*Cottunculus* spp.	2.19	1.83	2.08	10.46	35	7
*Icelus* spp.	1.16	0.87	0.98	2.90	14	7
*Leptagonus decagonus*	1.14	0.48	1.93	23.08	209	7
*Anarhichas lupus*	2.74	2.22	2.54	9.71	41	8
*Hippoglossoides platessoides*	1.89	1.10	2.36	20.00	655	8
*Lycodes gracilis*	0.99	0.57	1.21	6.15	139	8
*Melanogrammus aeglefinus*	1.07	0.73	1.22	12.57	2527	8
*Pleuronectes platessa*	0.68	0.40	0.83	3.58	27	8
*Anarhichas minor*	1.67	1.47	1.62	7.53	40	9
*Lycodes esmarkii*	0.95	0.56	0.87	3.41	27	9
*Anisarchus medius*	0.06			0.06	1	
*Enchelyopus cimbrius*	0.15		0.06	0.23	3	
*Gaidropsarus argentatus*	0.87		0.46	1.52	4	
*Glyptocephalus cynoglossus*	2.60			2.60	1	
*Gymnocanthus tricuspis*	2.32		2.63	6.67	5	
*Lampanyctus macdonaldi*	0.91			0.91	1	
*Lophius piscatorius*	2.85		1.40	3.85	2	
*Lycodes eudipleurostictus*	0.93			0.93	1	
*Lycodes polaris*	0.40			0.40	1	
*Lycodes seminudus*	1.18		1.41	3.56	5	
*Myoxocephalus scorpius*	1.78			1.78	1	
*Phycis blennoides*	0.83			0.83	1	
Grand Total	1.98	1.07	2.64	33.51	17,871	

*Note*: The last column shows cluster association from Eriksen et al. (2020). Also included in the lower part of the table are values for 12 additional species of fish with sample size 5 or less.

The sequence of species in Table 3 has been arranged according to trophic clusters obtained by Eriksen et al. (2020; see also Eriksen et al., 2024). Cluster 1 is a group of predominantly piscivorous species. Clusters 2–4 are planktivores (2, euphausiids and copepods; 3, hyperiid amphipods; 4, gelatinous plankton), while clusters 5–9 are benthivores (5, worms; 6, large demersal crustaceans plus fish; 7, small demersal crustaceans; 8, mixed benthos; 9, echinoderms). Fish from cluster 3 had the highest mean and median stomach contents, followed by fish belonging to clusters 1 and 4 (Figure S2).

### 3.5 Patterns of ranked stomach content from highest to lowest on log scale

**FIGURE 4:**
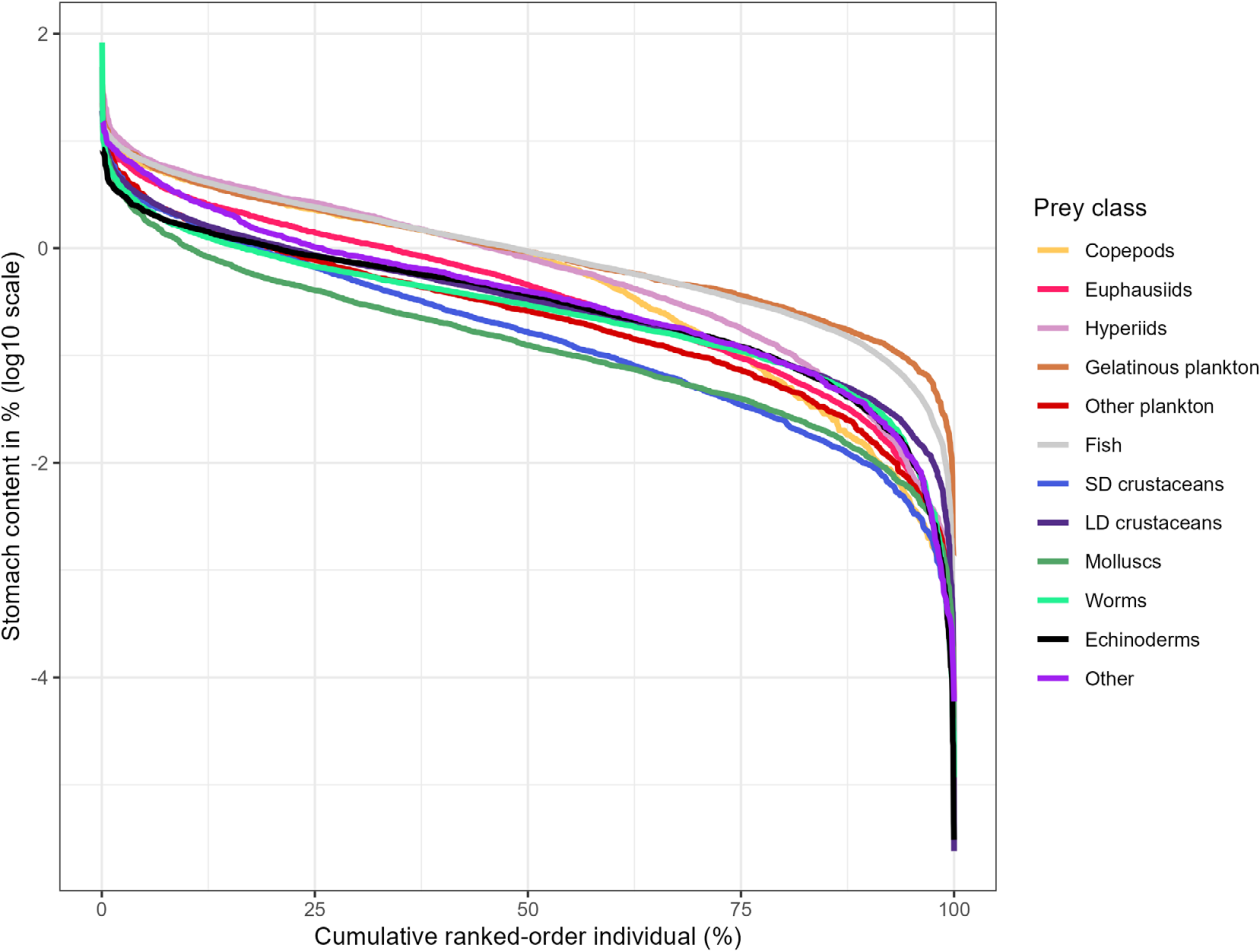
Plots of stomach content (% weight, log10‐transformed) for individual stomachs in ranked order from highest to lowest for different prey categories. Based on data from the full set of stomachs with food (*n* = 17,873). The number of individuals (stomachs) in ranked order on the *x* axis is shown on a relative scale as percentage. The numbers of stomachs containing each prey category are given in Table 2.

When the same data shown as frequency histograms in Figures 2 and S1 are plotted as individual stomachs in ranked order from highest to lowest, they reveal a common pattern for all prey categories as well as total stomach content. The plots in Figure 4 are with log10‐transformed weight of stomach content plotted versus stomach number in decreasing order. The curves show a log‐linear (or near log‐linear) segment between log10 values of ~0.5 and ~−1.0, with an upward curvature in the high end and a downward curvature in the low end. The two log10 values correspond to ~3% and 0.1% stomach content by weight. The upward curvature in the high end demonstrates that only a few stomachs had high weight of stomach content, for example >3–10%. The downward curvature in the low end demonstrates that relatively few stomachs had very low stomach content, for example less than log10 value −2 (<0.01%). The position of the curves on the *y* axis reflects what is shown in Figure 1 for mean and median values, with generally higher stomach contents of the plankton prey categories compared to benthic prey.

### 3.6 Rank distribution of stomach content weight for species and families of fish predators

**FIGURE 5:**
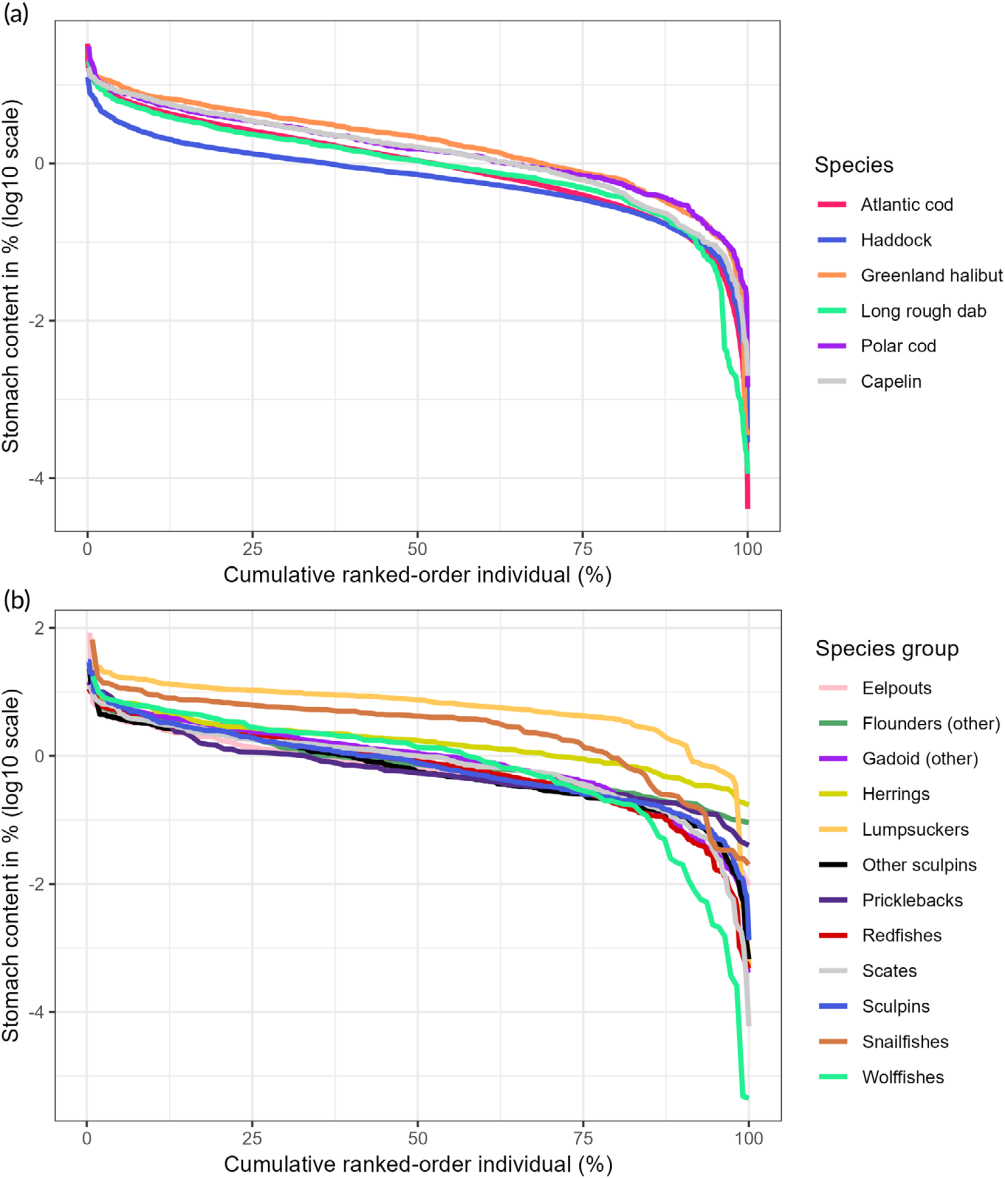
Plots of total stomach contents (weight‐% on log10 scale) of individuals in rank order from highest to lowest for (a) six species and (b) 12 families (or group of families) of fish predators. “Other gadoids” are species of family Gadidae (excluding Atlantic cod, haddock, and polar cod), and rocklings of family Lotidae. “Other flounders” are family Pleuronectidae excluding Greenland halibut and long rough dab. Sculpins are family Cottidae, while “other sculpins” are families Agonidae and Psychrolutidae.

The distribution of weight of total stomach content ranked from highest to lowest showed similar patterns for the six commercial species (including polar cod) with highest sample size (*n* = 609–8969) (Figure 5a) and 12 families (or groups of families) of fish predators (Figure 5b) to those shown for the various prey categories across the whole sample set (Figure 4). This pattern was an upward curvature to high stomach content in a small number of stomachs, a near log‐linear segment over most of the range of stomachs, and a downward curvature to very low values, again in a small number of samples. Greenland halibut among the species and lumpsuckers at the family level had the highest stomach content, while haddock among the species and eelpouts at the family level had generally lowest stomach content (Tables S1 and S2).

Maximum stomach content varied between the fish families apparently related to differences in body shape and stomach size and capacity. The highest maximum stomach content (30%–33% of body weight) was observed in the families Gadidae, Liparidae, and Cottidae, with somewhat lower values (23%–27%) in the families Pleuronectidae, Cyclopteridae, and Agonidae (see Table 3). The families Zoarcidae and Lotidae showed the lowest values of maximum stomach content (4%–7%). In addition, there were differences in maximum stomach content between species from different trophic groups within some of the families. Among the flounders (Pleuronectidae), predatory species with large mouths (Greenland halibut and long rough dab) had much higher maximum stomach content than benthivorous species with small mouths (plaice, dab), 20%–27% of body weight versus 0.7%–6.5%. A similar pattern was observed for cod (Gadidae), where predatory species (Atlantic cod) had the highest values (33.5%), while the maximum stomach content of benthivore species was lower (9%–14%).

### 3.7 Frequency distribution of stomach content for species of fish predators and prey groups

**FIGURE 6:**
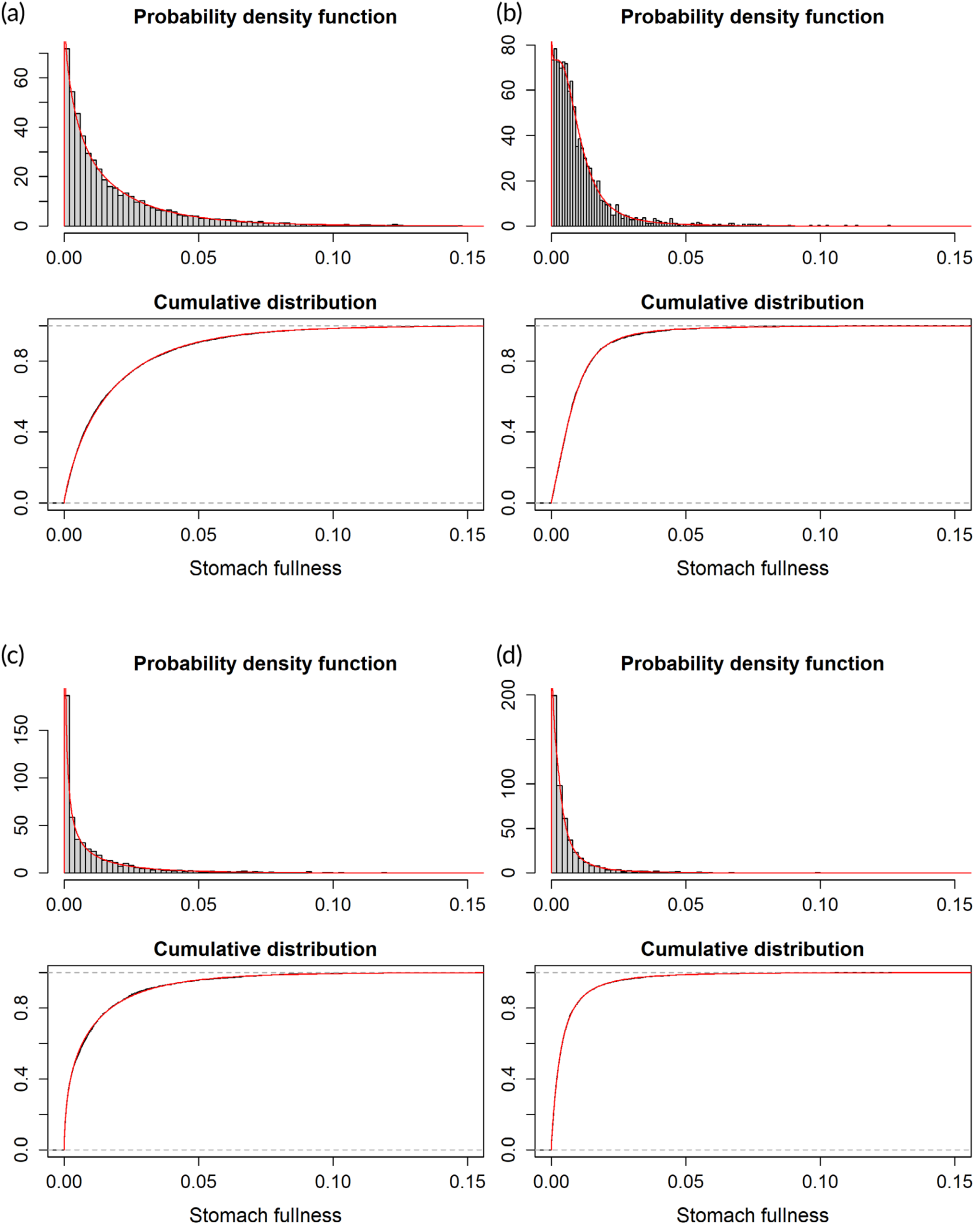
Frequency histograms with fitted distribution models (red lines) for data on total stomach contents for (a) Atlantic cod and (b) haddock, and for stomach contents of the prey groups (c) euphausiids and (d) worms for all species with stomachs containing such prey. The lower of the two panels for each case shows the cumulative version for both data (black line) and the fitted model (red line). The model is in each case the best‐fit model from applying the function *fitDist()* in R (see Table 4). Note that the model fits the data very closely and the two lines overlay each other.

The ranked plots for the 12 prey types and six selected species of fish predators in Figures 4 and 5a can be represented as frequency distributions by summing the number of individuals in bins of stomach content and switching the *x* and *y* axes. The frequency distributions and associated probability density functions (pdf) are shown for two species (cod and haddock, predominantly piscivore and benthivore, respectively) and two prey types (euphausiids and worms, plankton and infauna benthos) in Figure 6. Similar plots for the other four species and 10 prey types are shown in Figure S3.

When fitting distribution models (pdfs) to the data with the gamlss package in R, the Box–Cox power exponential model gave the best fit in eight of the 18 cases (Table 4). Two other versions of the Box–Cox model (Cole and Green, and *t*) gave the best fit in five more cases. Other models that came out best were the generalized inverse Gaussian model in three cases, and gamma or generalized gamma models in two cases (Table 4).

**TABLE 4:** Best model with parameter estimates when fitted to data on weight of stomach content (weight‐%) for six species of fish predators (total stomach content) and 12 types of prey across all species with stomachs containing each type of prey.

	Best model	Parameters			
		Mu	Sigma	Nu	Tau
*Species*					
Atlantic cod	Box–Cox power exponential	−4.493	0.289	0.232	0.784
Haddock	Box–Cox t	−5.525	0.574	0.878	0.851
Greenland halibut	Gamma	−3.477	0.057		
Long rough dab	Box–Cox power exponential	−4.739	0.664	0.506	0.137
Polar cod	Box–Cox Cole and Green	−4.142	0.152	0.187	
Capelin	Generalized inverse Gaussian	−3.682	1.598	0.813	
*Prey types*					
Copepods	Generalized inverse Gaussian	−4.154	2.090	0.388	
Euphausiids	Box–Cox power exponential	−5.489	0.588	0.161	1.005
Hyperiids	Generalized inverse Gaussian	−3.944	2.426	0.483	
Gelatinous plankton	Box–Cox power exponential	−4.647	0.259	0.138	0.898
Other plankton	Generalized gamma	−5.622	0.523	0.358	
Fish	Box–Cox power exponential	−4.626	0.322	0.190	0.874
SD crustaceans	Box–Cox power exponential	−6.393	0.684	0.090	0.988
LD crustaceans	Box–Cox Cole and Green	−5.691	0.407	0.122	
Molluscs	Box–Cox Cole and Green	−6.653	0.566	0.064	
Worms	Box–Cox power exponential	−5.832	0.404	0.167	0.422
Echinoderms	Box–Cox t	−7.018	1.511	0.626	1.766
Other	Box–Cox power exponential	−5.531	0.549	0.136	0.561

*Note*: The number of estimated parameters varies between models from two to four. The names of the parameters are taken from the *fitDist()* R function outputs. See Figures 6 and S3.

Abbreviations: LD, large demersal; SD, small demersal.

The frequency distributions of stomach content were clearly logarithmic in nature but differed from a log‐normal distribution by being more skewed to the left (right‐leaning; Figure S4), reflected by negative skewness values with log‐transformed data for the six species (from −0.9 to −2.0; Table S1). The distributions also showed positive excess kurtosis (values from 1.3 to 5.9; Table S1), reflecting the tail in the low end of the log distributions (Figure S4). The log‐transformed data for the 12 groups of species by families showed similar features in their frequency distributions (not shown), leaning to the right with negative skewness values (−0.1 to −3.6; Table S2). While the log‐normal distribution was not identified as the best model in any of the cases tested (Table 4), it gave a reasonable fit to the data on total stomach content for the six selected species as well as the 12 prey categories (Figure S3).

### 3.8 Weight of stomach content in relation to body size

**FIGURE 7:**
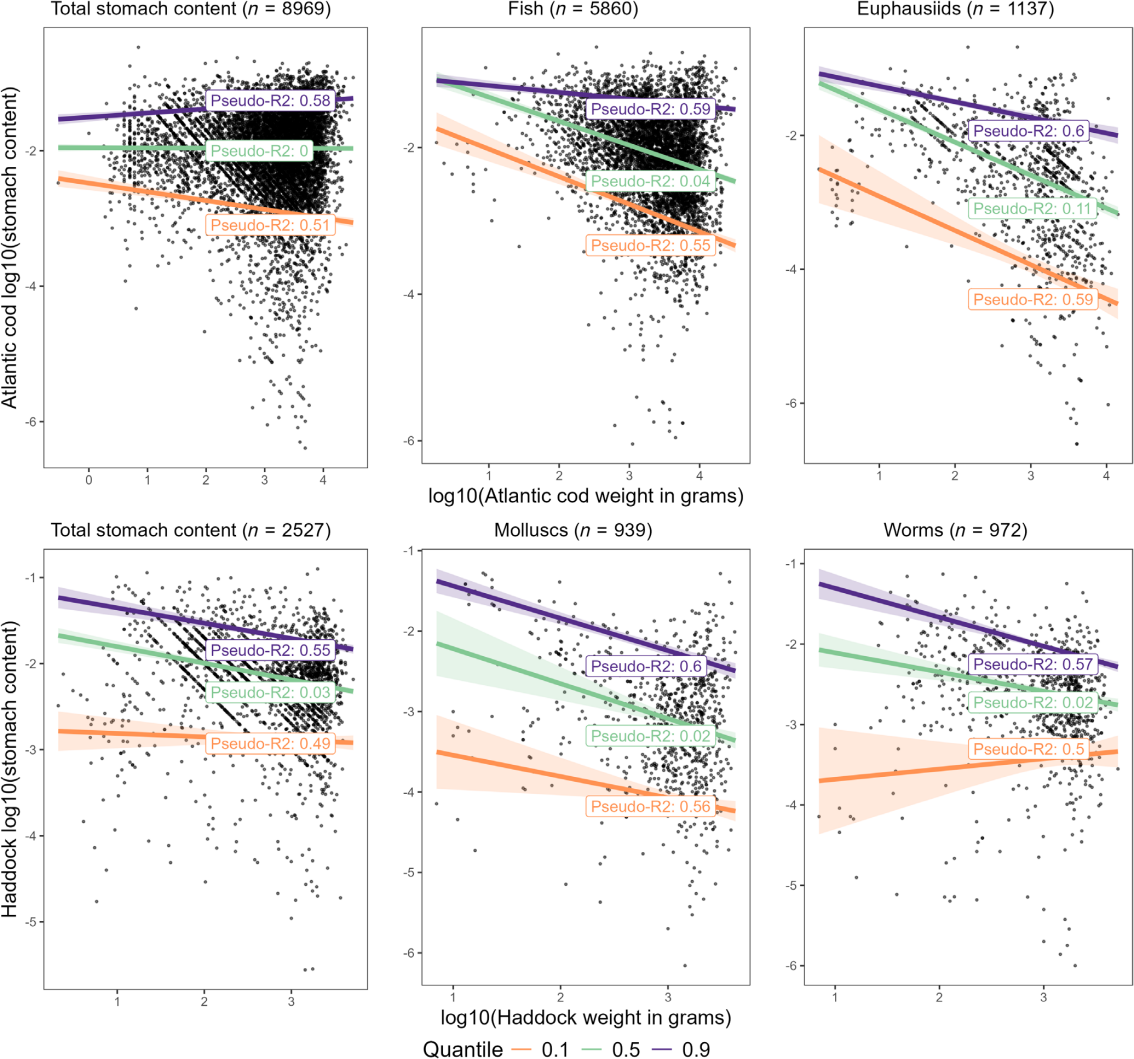
Stomach content (weight of prey per weight of predator fish) versus individual size (weight) of fish predators for Atlantic cod (upper row) and haddock (lower row). The three panels for each species show total stomach content, and contents of fish and euphausiid prey for cod, and contents of mollusc and worm prey for haddock. The data are log10 transformed. The upper (violet) and lower (orange) lines are quantile regression lines for upper (90%) and lower (10%) quantiles, while the middle green line is the overall (median) regression. The shaded bands are 95% confidence bands around the regression lines.

We have examined individual variation in stomach content (weight per fish predator weight) versus the size (weight) of the fish predators for the six selected species with the largest sample sizes. The variation in diet composition at the individual level for the six species is illustrated with box‐whisker diagrams in Figure S5. The individual total stomach content varied by four to five orders of magnitude, and scatter plots revealed that the size of the individuals explained only a small fraction of the variance in either total stomach content or in dominant diet components for each of the six selected species (Figures 7, S6, and S7). The upper quantile regression line (90%) showed a decreasing trend in most cases for the piscivorous species (Atlantic cod and Greenland halibut) and benthivorous species (haddock and long rough dab) (Figures 7 and S6). For the planktivores (capelin and polar cod), results were more variable and uncertain (Figure S7).

The scatter of data points and the lower quantile (10%) regression line demonstrate a conspicuous pattern of increased occurrence of low stomach content with increasing body size, seen most clearly for the larger species such as Atlantic cod and Greenland halibut (Figures 7 and S6). The overall (median) regression showed declining trends with increasing body size for Atlantic cod, haddock, Greenland halibut, and long rough dab (Figures 7 and S6), while there were increasing trends for the smaller planktivores (polar cod and capelin; Figure S7). It is noteworthy that the median regression explained just a few percent of the total individual variance in weight of stomach content for either total content or contents of specific prey categories. The case with the highest fraction of variance explained (*R* ^2^ = 0.26) was seen for Greenland halibut with hyperiid prey (Figure S6). Despite the low fraction of variance explained, many of the median regression trends were highly significant, seen from the narrow confidence bands. This reflects the high sample size, which makes even small effects statistically significant.

The low fraction of variance explained by size was confirmed with a Random Forest analysis of the individual data of total stomach content for the full data set for all species (*n* = 17,873 individual stomachs). The variables “species” and “sampling station” (individual trawl samples) were the most important, but along with body size, season, and geographical area they explained only 22% of the total variance in individual stomach content (*R* ^2^ = 0.22; Table S3).

## 4 DISCUSSION

### 4.1 Quantitative stomach content follows a common and consistent pattern

Quantitative stomach content analysis can tell us what fish are eating and how much they are consuming. This may inform how species of fish and their prey are connected in food webs, which in turn may contribute to ecosystem analysis, modelling, and improved understanding. We have in previous papers on the 2015 stomach data set from the Barents Sea examined trophic patterns across species, length groups, geography, and seasons (Eriksen et al., 2020, 2021, 2024). These studies used average stomach content across individuals at the species level. Here, we are focusing on the data in more detail by examining the individual variation within species. The main finding is a common and consistent pattern in the individual variation. The pattern appears to be general and robust, and shows up in data aggregated across species as well as in data within species for both total stomach content and contents of specific prey types (e.g., plankton or benthos), and for various trophic types of fish (e.g., planktivores, benthivores, and piscivores). The difference in sampling protocols between commercial and non‐commercial species appears to have had no noticeable effect on this basic result.

The pattern is illustrated with ranked plots of the weight of stomach content (log transformed) across the individuals of groups of fish predators as shown in Figures 4 and 5. The ranked plots are related to frequency distributions (histograms) and pdfs, with the latter showing the number of individuals (or proportions or density) within intervals of stomach content. The frequency distributions or pdfs are usually shown with the *x* and *y* axes reversed compared to the ranked plots in Figures 4 and 5. The best distribution models gave very good fit to the data on stomach content (on linear scale), as illustrated in Figures 6 and S3. In most cases (13 out of 18), a variant of the Box–Cox model (power exponential, Cole and Green, or *t*) was identified as the best model (Table 4). The Box–Cox model is flexible, with four (or three in the case of the Cole and Green version) parameters in the equations (including parameters reflecting skewness and kurtosis) (Table 4). Other models that came out as best in some cases were the “generalized inverse Gaussian” and the “generalized gamma”, which are three‐parameter models. The gamma model was selected as the best in one case (Greenland halibut). A gamma model was used by Stefánsson and Pálsson (1997) to model the stomach content of Atlantic cod in Icelandic waters. It is a two‐parameter but flexible and versatile model.

The good fit of models such as Box–Cox and others to the quantitative stomach content data for both prey types and predator fish species demonstrates that mathematical descriptions of the data are feasible for purposes where such descriptions can be useful (e.g., modelling of trophic interactions). However, the models may be difficult to interpret in mechanistic terms. This is because the use of three or four parameters, which allows great flexibility and good fit to data, at the same time complicates interpretation.

The log‐normal distribution was not identified as the best model in any of the 18 cases examined with data (six species and 12 prey types; Table 4). However, the log‐normal distribution gave a reasonable approximation for the data of the six selected data‐rich species as well as the 12 prey types (Figure S3), although we show that the distribution was somewhat skewed (right‐leaning, or over‐dispersed) compared to the log‐normal distribution (Figure S4). That the individual variation in stomach content is log‐normal‐like is perhaps surprising, but it offers a basis for interpretation in terms of the underlying feeding processes. In the following, we discuss various aspects of the distribution of the quantitative stomach contents. First, we consider the general shape of the distribution: why is it log‐normal‐like, and what does this reveal about feeding ecology? We then go on to consider the upper and lower ends of the distribution. The upper end is a tail of high values on the log scale and apparently challenges the concept of a “full stomach”. The lower end is the transition to the relatively large fraction of empty stomachs in the sampled material. Finally, we discuss what the high proportions of fish with empty stomachs and the generally low median and mean stomach content mean for trophic ecology.

### 4.2 The log‐normal‐like shape of individual variability in weight of stomach content

A normal distribution (Gaussian bell‐shaped) indicates additive effects of several factors according to the central limit theorem (CLT) (Lyon, 2014). A log‐normal distribution is by the same reasoning (applying the CLT) indicative of multiplicative effects of factors (since the log of a product is the sum of the log of the separate factors) (Limpert et al., 2001). The log‐normal distribution of individual stomach content (although somewhat skewed) therefore suggests that the foraging, which results in stomach content, is reflecting the multiplicative role of factors that influence or shape the outcome of feeding. Feeding is itself a complex process affected by many factors on different scales. This includes life history, choice of habitat, and the distribution, abundance, and behavior of both prey and predators. Predators are here the foraging fish themselves, but also their own predators that shape the feeding behavior of fish. The process of feeding consists of several stages, such as searching for prey, encountering prey, and then attacking, handling, and, if successful, ingesting the prey (Holling, 1959).

Trophic interactions are usually modelled as a multiplicative process. Thus, the classical Lotka–Volterra equations for predator–prey interactions include the product of abundances or densities of predator and prey. Likewise, feeding rate is commonly modelled as a product of the probabilities of encounters, elicited attacks, and successful capture, handling, and ingestion.

The general shape of the frequency distributions of individual stomach contents was similar for fish predators of different lifestyles, habitat associations (benthic, demersal, pelagic), and trophic types (planktivores, benthivores, piscivores), and similar also across species for different prey types (e.g., groups of plankton or benthos). This suggests that the underlying processes and factors are fundamentally similar, with a general outcome in terms of similar statistical distributions of weight of stomach contents. This in turn has implications for the treatment, presentation, and use of stomach content data for subsequent statistical analyses, where simple summary statistics of a group of individuals (i.e., mean and standard deviation of stomach content) should include information on any strong deviation from a normal distribution for linear‐scale data.

### 4.3 Maximum stomach content and the concept of “full stomach”

The maximum stomach content was typically 15%–30% (relative to the individual weight of the fish) for most of the prey categories, with an overall maximum value of 33.5% for total stomach content recorded in the 17,873 stomachs investigated (note that two higher values were removed as outliers). A value of ~30% is probably a physical maximum among the fish species included in our study (see, e.g., Burley & Vigg, 1989; Jobling et al., 1977; and Phelps et al., 2007, for determination of maximum stomach size). The maximum stomach size (corresponding to the maximum stomach content) is dependent on the anatomy of the species, especially the shape of the fish and size of the belly region, and the size and distensibility of the stomach. Extreme anatomical adaptation is found for some species living in the deep ocean, where black swallower (*Chiasmodon niger* Johnson 1864) in the family of swallowers (Chiasmodontidae) can swallow fish prey twice their own length and ~10 times their weight (Jordan, 1905).

The highest maximum value of 33.5% was found for an individual of Atlantic cod, which is a piscivore with a deep belly and a large and distensible stomach. This allows it to swallow large prey. When feeding cannibalistically on other cod, or on haddock, which are common prey for cod in the Barents Sea, cod swallows prey up to about 50% of their own length (Holt et al., 2019). One such large fish prey would represent a stomach content of about 13% (on weight‐basis) calculated from the weight–length relationship for cod (Jørgensen & Fiksen, 2006). Other species with maximum stomach content of ~30% were Greenland halibut, polar cod, leatherfin and Atlantic spiny lumpsucker, bigeye sculpin, Atlantic hookear sculpin, and the snailfish *Careproctus* spp. (Table 3). The two lumpsuckers and *Careproctus* are rather globular in shape, while the two sculpins are species with a large head and broad body shape (see Mecklenburg et al., 2018 for drawings and photos of the species). Greenland halibut is mainly a piscivore at large size (Dolgov, 2016; Eriksen et al., 2020, 2024), with a relatively large head and mouth gape. Another species of flounder, long rough dab, had maximum stomach content of 20%. Polar cod, which is a planktivore (feeding predominantly on the amphipod *Themisto*, while larger specimens prey also on fish), resembles Atlantic cod, with a relatively large head and apparently a similar high stomach capacity. Haddock, in contrast, which is mainly a benthivore, had maximum stomach content of 13%. Capelin, which is a dominant planktivore in the Barents Sea (feeding on copepods and euphausiids; Dolgov, 2016; Eriksen et al., 2020, 2024), had maximum stomach content of 17%. Capelin is a slender species with probably less room for a large stomach compared for instance to polar cod.

Maximum stomach content for the species was correlated with both mean stomach content and sample size (Figure 3). Both correlations can be interpreted to reflect the common shape (skewed log‐normal) of the stomach content versus ranked individuals (Figures 4 and 5). From the common shape, one can expect a relation between the overall level of the distribution (reflected by the mean) and the level in the high end. The high end of the distribution is related to the tail‐end of the log‐normal distribution on the right (high) side and is characterized by high variability. At the same time, observations at the high end are rare, and they reach into a zone of statistical outliers. Thus, with more samples, one is more likely to record high values. This is probably the explanation for the increase in maximum stomach content with increased sample size shown in Figure 3b.

The log‐tail of high values of stomach content for species (or groups of species) is somewhat surprising and challenges the concept of “full stomach”. As we have discussed already, there is likely to be a physical maximum stomach size, which might appear as a truncated upper end of the frequency distribution (pdf). This is not seen in the empirical data, although there may be a tendency to truncated distributions for capelin and Greenland halibut among the species with high sample size (Figure S4c,e). The functional response of feeding rate of a predator versus density of prey is commonly modelled as some form of asymptotic function, for example the Ivlev equation (Ivlev, 1955) or the various Holling types of functional response (Holling, 1965). The mechanistic basis for asymptotic functions lies in the maximum number of prey that can be handled and ingested (this number is inversely related to handling time), and to the satiation level of the predator, basically that it gets “full” and has no more room for another prey. A priori, we would expect to see the “full stomach” as an asymptote for a truncated frequency distribution in the high end. The fact that this does not appear to be the case suggests that a full stomach is a rare event. “Full stomach” would here mean filled to its maximum capacity. In our large data set of fish stomach contents from individuals collected with trawl sampling in offshore areas of the Barents Sea, we may occasionally see stomachs with maximum content (~30%), but these are rare events and form part of a log‐normal high tail.

### 4.4 Empty stomachs

About a third of the total number of sampled stomachs in the 2015 data set were empty (Figure 2). Eriksen et al. (2020) presented information on the number (fraction) of empty stomachs across the investigated species. Of the six selected species with high sample number, the two flatfish species (Greenland halibut and long rough dab) had the highest proportions of empty stomachs (72% and 58%), while cod and haddock had the lowest proportions (22% and 24%). For cod in the Barents Sea, a large data set of 400,000 stomachs collected over many years (1930–2018, of which our data for 2015 is a part) had a fraction of 26% empty stomachs, while an even larger Russian data set of more than 3 million stomachs (with qualitative stomach analysis) had 22% empty stomachs (Townhill et al., 2021). A compilation of information from other extensive stomach sampling programs shows that the fraction of empty stomachs is generally large, often 20%–40% or more (Table 5).

**TABLE 5:** Fraction (%) of empty stomachs in some published studies in various marine ecosystems based on extensive stomach sampling programs.

Location	Fish community	Empty stomachs (%)	Reference
Barents Sea	Atlantic cod	25.5	Townhill et al. (2021)
Barents Sea	Haddock	30–40	Antipova et al. (1990)
Barents Sea	Long rough dab	69	Simacheva & Glukhov (1990)
Barents Sea	Demersal, pelagic, 60 species	2–86	Dolgov (2016)
Baltic Sea	Atlantic cod	17–26	Patokina & Nigmatullin (2017)
Celtic Sea	Demersal, pelagic, 5 species	6–65	Pinnegar et al. (2003)
NW Atlantic, US shelf	Benthic flatfish, 9 species	13–48	Link et al. (2002)
Puget Sound, NE Pacific	Demersal, 21 species	64.5	Reum & Essington (2008)
Tasmania	Demersal, pelagic, 15 species	7–62	Blaber & Bulman (1987)
Russian Far East (Bering Sea, Sea of Okhotsk, Japan Sea)	Demersal, pelagic, 79 species	0–91	Chuchukalo (2006)
Antarctic waters	Demersal, pelagic, 22 species	2–75	Tarverdieva, Kozlov, et al. (1996), Tarverdieva, Podrazhanskaya, & Pinskaya (1996)

The tail in the low end of the skewed log‐normal distribution (Figure S4), corresponding to the curved lower end of the ranked stomach content plots (Figures 4 and 5), represents the transition to empty stomachs. This transition is characterized by relatively few individuals with very low recognizable and measurable stomach content. There is a method caveat here in that a single (or a few) small prey may be more likely to be found in a large stomach than in a small. Thus, in plots of stomach content versus weight of individual predator fish, the lower part of the distribution showed a pronounced decrease in stomach content versus size of the fish (Figure 7). An example helps to understand this feature of the data. A 2‐cm long krill (*Thysanoessa inermis*) weighs ~100 mg wet weight (Dalpadado & Skjoldal, 1991). One individual of such krill would make up 10^−5^ (0.001%) of a 10‐kg cod, and 10^−3^ (0.1%) of a cod weighing 100 g. This corresponds quite well to the lower values seen for cod in Figure 7.

Recognizable stomach content is in the form of individual prey items. An interpretation of the sharp drop‐off into low stomach content at the low‐end of the distribution is that it reflects a “thinning” of observations related to the minimum size of prey items and the frequency that such items are encountered in stomachs dependent on the size of the fish predator (and the size of the stomach). The “quantum” nature of prey (individuals of similar size from cohorts of prey species) may also be the explanation for the striped patterns seen in some of the plots of stomach contents versus size of the predator fish, seen most clearly in plots for polar cod and capelin (Figure S7). One or some small number of uniform prey would represent decreasing weight proportion with increasing weight of the fish predators.

### 4.5 Implications of the high frequencies of empty stomachs and stomachs with low food content

We have seen that the weight of stomach content of fish predators follows a general right‐skewed (log‐normal‐like) statistical distribution, and that “full stomachs” are rarely encountered, even in a large sample collection such as our data from 2015. The mean stomach content for the various prey groups was typically 0.5%–2% on weight basis (for stomachs containing food) (Figure 1), while the mean total stomach content for the 55 species was 2.3% (standard deviation = 2.4, Table 3). However, the distribution among species was skewed and approximately two‐thirds of the 55 species (with sample size >5 stomachs) had mean total stomach content between 0.5 and 2.5% (Table 3). The skewness in distribution was reflected in median stomach content being roughly half the mean values (Figure 1 and Table 3). Thus, both mean and median values revealed generally low stomach content for the sampled populations of fish in the Barents Sea.

**FIGURE 8:**
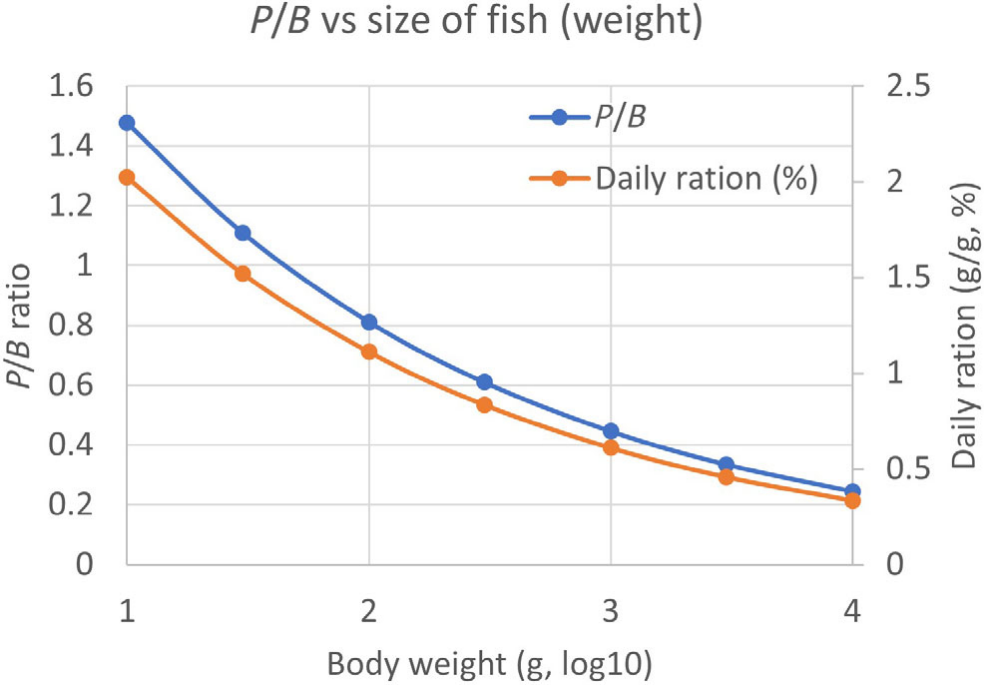
Annual *P*/*B* ratio and estimated food demand as daily ration for fish of body weight at maturity from 10 g to 10 kg. The *P*/*B* ratio is calculated from the empirical equation for fish in Banse and Mosher (1980). The annual food demand is calculated from the *P*/*B* ratio, assuming 20% gross growth efficiency [multiplying annual production (*P*) by 5; Skjoldal et al., 2004] and expressed as daily ration by dividing the annual value by 365 days.

A simple explanation for the generally low stomach content, including the high proportion of empty stomachs, is that the fish eat what they need to balance their energy requirements, which is met by a relatively low daily ration. The fishes we have sampled from the wild evidently do not feed ad libitum to fill up their stomachs like fish in captivity can if offered an abundant supply of food. So, how much food is required for the Barents Sea fish? We illustrate this by a simple bioenergetic calculation. Banse and Mosher (1980) provided an equation for the annual *P*:*B* ratio versus body size for fish, where *P* is the annual production and *B* is the annual average biomass for a population. The empirical equation was a power function (log[*P*/*B*] = 0.44–0.26*log *M*, where *M* is adult body mass in units of kcal), like the allometric equations for rates of metabolism or growth versus body size (Andersen et al., 2016). Solving the equation for size of fish from 10 g to 10 kg wet weight (converted from kcal) gives a *P*/*B* ratio falling from 1.5 at 10 g to 0.25 at 10 kg body weight of fish (Figure 8). From this relationship, capelin would have an annual *P*/*B* ratio of 1.1 (weight at maturity ~20 g; Froese et al., 2014; Gjøsæter, 1998), while cod would have a ratio of ~0.3 (weight at maturity ~3 kg, Yaragina et al., 2011).

The annual production (*P*) (relative to the weight of the fish) was converted to annual food demand by assuming 20% gross growth efficiency (Skjoldal et al., 2004). Expressed as daily ration (by dividing by 365), the food demand would decrease from 2% per day for a 10‐g fish to 0.3% per day for a 10‐kg fish (Figure 8). This range is comparable to the ranges of mean and median values of stomach content for most of the species of predator fish in Table 3. Thus, the mean stomach content across the individuals of a species is of the same order as the daily food demand. For a more detailed comparison, the turnover of food in the stomach due to digestion needs to be taken into account. Digestion of food depends on the anatomy and physiology of the digestive tract of the fish predators, and on the digestibility of the prey including prey size, as well as on predator weight and water temperature. The turnover of stomach content, or the rate of gastric evacuation, is often modelled as a function of the stomach content itself (Andersen, 2022; Jobling, 1981; Karamushko, 2007; Salvanes et al., 1995). In experiments with plaice (*Pleuronectes platessa* L. 1768) and European flounder (*Platichthys flesus* L. 1758) at temperatures of 1–10°C, full digestion of prey (fish, shrimp, bivalve) took 1–3 days (Karamushko, 2007).

For a species like Atlantic cod, with a weight of ~3 kg at maturation (Yaragina et al., 2011), studies in the Barents Sea have found daily food rations to be of order 1% relative to body weight (Ajiad & Korsbrekke, 1992; Bogstad & Mehl, 1997; Dolgov et al., 1992). Under experimental conditions, the daily maintenance rations for cod were 0.7%–1.0% of body weight for individuals of size between 100 g and 1 kg, while the maximum daily ration was 3.7% (Karamushko, 2007). The comprehensive stomach sampling program is used to calculate the total annual food consumption by the Barents Sea cod stock (Bogstad et al., 2015; Bogstad & Mehl, 1997; Holt et al., 2019). The average annual food consumption for the 15‐year period 2006–2020 was ~6 million tonnes (varying from ~5 to 8 million tonnes) when the cod stock was at a high level of ~3 million tonnes (range 1.5–4.4 million tonnes; WGIBAR, 2021 [see figures 3.7.4 and 4.2.1]; Kjesbu et al., 2023). This gives an average daily ration of ~0.5% per unit body weight. Similar results with daily ration varying around 0.5–1% were obtained for various age groups of Baltic Sea cod (Funk et al., 2023).

A clear pattern in our results was higher stomach content of planktonic prey compared to benthic prey (Figure 1). This probably reflected higher food demand by planktivorous fish, which are typically small species with relatively high rates of growth. In contrast, small benthivores in the groups of, for example, sculpins and eelpouts are relatively slow‐growing species and therefore presumedly need less food compared to planktivores.

## 5 CONCLUSION

Stomach fullness (weight of stomach content per weight of fish predator, g g^−1^ as %) varied by several orders of magnitude within species or groups of species. The maximum stomach content was typically ~15–30% for most species, but high stomach content (e.g., >10%) was rarely observed. The low stomach content graded into a large fraction of empty stomachs (35% across all sampled individuals). Between the maximum and empty stomachs, the individual variation in weight of stomach content followed a common and consistent statistical distribution that was right‐skewed and log‐normal‐like. The distribution was generally modelled well with variants of the Box–Cox, generalized inverse Gaussian, or gamma models. The common statistical pattern was relatively consistent among species and trophic types of fish predators (e.g., piscivores, planktivores, and benthivores), and across different types of prey (e.g., groups of plankton or benthos).

Only a small fraction (22%) of the total variance in the weight of individual stomach content was explained by the variables “species”, “sampling station”, “individual size”, “geographical area”, and “season”. The consistency of the statistical pattern and the high fraction of unexplained variance suggest that the strict statistical distribution across the wide range of stomach content has a general biological basis. As such it is an inherent property of fish populations, reflecting some common and overarching principles governing their foraging activity. The fact that the common pattern is log‐normal‐like suggests that feeding is a multiplicative process involving a complex of factors, including level of satiation, life‐history traits, habitat association, and the distribution, abundance, and behavior of both prey and predators.

We believe this study provides food for thought for how to extract more information on the underlying processes of fish feeding from the statistical pattern of stomach content. On a practical level, it may guide further refinement of stomach sampling programs to monitor predator–prey interactions, which is an important aspect to support the ecosystem approach to management. It will also be of value in the further development of analytical approaches to the study of trophodynamics involving fish in various marine ecosystems.

## AUTHOR CONTRIBUTIONS

E.E., A.D., and H.R.S. designed the research. H.R.S. and K.O. analyzed the results. H.R.S. wrote the manuscript. All authors revised the manuscript and agreed with its publication.

## FUNDING INFORMATION

Research Council of Norway, Grant/Award Numbers 228880, 276730.

## Supporting information


**DATA S1.** Supporting Information.
