# Supplementary material for: Diet and trophic structure of fishes in the Barents Sea: between empty and full stomachs – large individual variability follows a common pattern
Source: J Fish Biol. 2025 Jan 17;106(6):1734–51. doi: 10.1111/jfb.16058 (PMC12244309; doi:10.1111/jfb.16058)
Supplement: Supplementary file 1 — DATA S1. Supporting Information. [file JFB-106-1734-s001.docx]

**Diet and trophic structure of fishes in the Barents Sea: between empty and full stomachs – large individual variability follows a common pattern**

Hein Rune Skjoldal, Elena Eriksen, Kotaro Ono, Andrey Dolgov

*Journal of Fish Biology*, Article DOI: 10.1111/jfb.16058

**Supporting information**

**Additional tables and figures**

Tables S-1 – S-3

Figures S-1 – S-7

**Tables**

Table S-1. Summary statistics of the data on total stomach content (% weight) for 6 species of fish predators. Kurtosis and skewness values are also shown for log10 transformed data.

|  | Cod | Haddock | Greenland halibut | Long rough dab | Polar cod | Capelin |
| --- | --- | --- | --- | --- | --- | --- |
| *Linear* |  |  |  |  |  |  |
| Mean | 1.95 | 1.07 | 3.13 | 1.89 | 2.62 | 2.52 |
| Median | 1.10 | 0.73 | 2.15 | 1.10 | 1.54 | 1.60 |
| Standard Deviation | 2.38 | 1.22 | 3.24 | 2.36 | 3.04 | 2.67 |
| Maximum | 33.51 | 12.57 | 27.78 | 20.00 | 30.77 | 16.50 |
| Kurtosis | 12.35 | 16.26 | 7.24 | 15.15 | 18.29 | 3.32 |
| Skewness | 2.71 | 3.30 | 2.09 | 3.23 | 3.27 | 1.74 |
| *Log10* |  |  |  |  |  |  |
| Kurtosis | 3.63 | 3.65 | 5.50 | 5.92 | 1.74 | 1.33 |
| Skewness | -1.34 | -1.28 | -1.62 | -2.02 | -0.86 | -1.02 |
| n | 8969 | 2527 | 712 | 655 | 609 | 1078 |

Table S-2. Summary statistics of data on total stomach content (% weight) for groups of species of fish predators by families. See legend to Fig. 5 for more information on families. Kurtosis and skewness values are also shown for log10 transformed data.

|  | Gadoid (others) | Herr-ings | Scul-pins | Other scul-pins | Eel-pouts | Snail-fishes | Lump-suckers | Prickle-backs | Wolf-fishes | Red-fishes | Floun-ders (other) | Scates |
| --- | --- | --- | --- | --- | --- | --- | --- | --- | --- | --- | --- | --- |
| *Linear* |  |  |  |  |  |  |  |  |  |  |  |  |
| Mean | 1.86 | 2.24 | 1.69 | 1.27 | 1.09 | 4.39 | 7.85 | 1.26 | 2.13 | 1.44 | 1.42 | 1.46 |
| Median | 1.15 | 1.71 | 0.83 | 0.57 | 0.62 | 3.77 | 7.50 | 0.67 | 1.35 | 0.86 | 0.74 | 0.99 |
| SD | 2.13 | 2.03 | 2.84 | 1.96 | 1.22 | 4.02 | 5.18 | 2.05 | 2.60 | 1.71 | 1.46 | 1.71 |
| Maximum | 14.57 | 11.59 | 30.00 | 23.08 | 6.87 | 32.14 | 27.00 | 16.67 | 17.59 | 11.32 | 6.56 | 12.85 |
| Kurtosis | 6.53 | 4.13 | 41.18 | 63.09 | 4.76 | 13.98 | 1.62 | 28.15 | 11.00 | 7.70 | 1.99 | 11.16 |
| Skewness | 2.18 | 1.90 | 5.35 | 6.40 | 2.06 | 2.63 | 1.00 | 4.73 | 2.61 | 2.39 | 1.57 | 2.79 |
| *Log10* |  |  |  |  |  |  |  |  |  |  |  |  |
| Kurtosis | 2.09 | -0.27 | 0.80 | 2.29 | 1.18 | 1.91 | 18.56 | 5.84 | 5.62 | 2.80 | -0.79 | 5.50 |
| Skewness | -1.26 | -0.24 | -0.50 | -0.98 | -0.41 | -1.41 | -3.57 | -1.55 | -2.16 | -1.43 | -0.13 | -1.90 |
| n | 653 | 124 | 595 | 253 | 222 | 157 | 201 | 132 | 110 | 350 | 75 | 262 |

Table S-3. Random Forest Analysis of total stomach content for all individuals in the total data set comprised of 66 species (n = 17,873). Importance of factors are ‘impurity corrected’ relative measures.

| Factors | n | | Importance |
| --- | --- | --- | --- |
| Species | 66 | 0.58 | |
| Sample (trawl haul) | 1401 | 0.58 | |
| Individual length |  | 0.21 | |
| Season | 4 | -0.12 | |
| Geographical area | 15 | -0.05 | |
|  |  |  | |
| Total variance explained |  | R^2^ = 0.22 | |

**Figures**

**
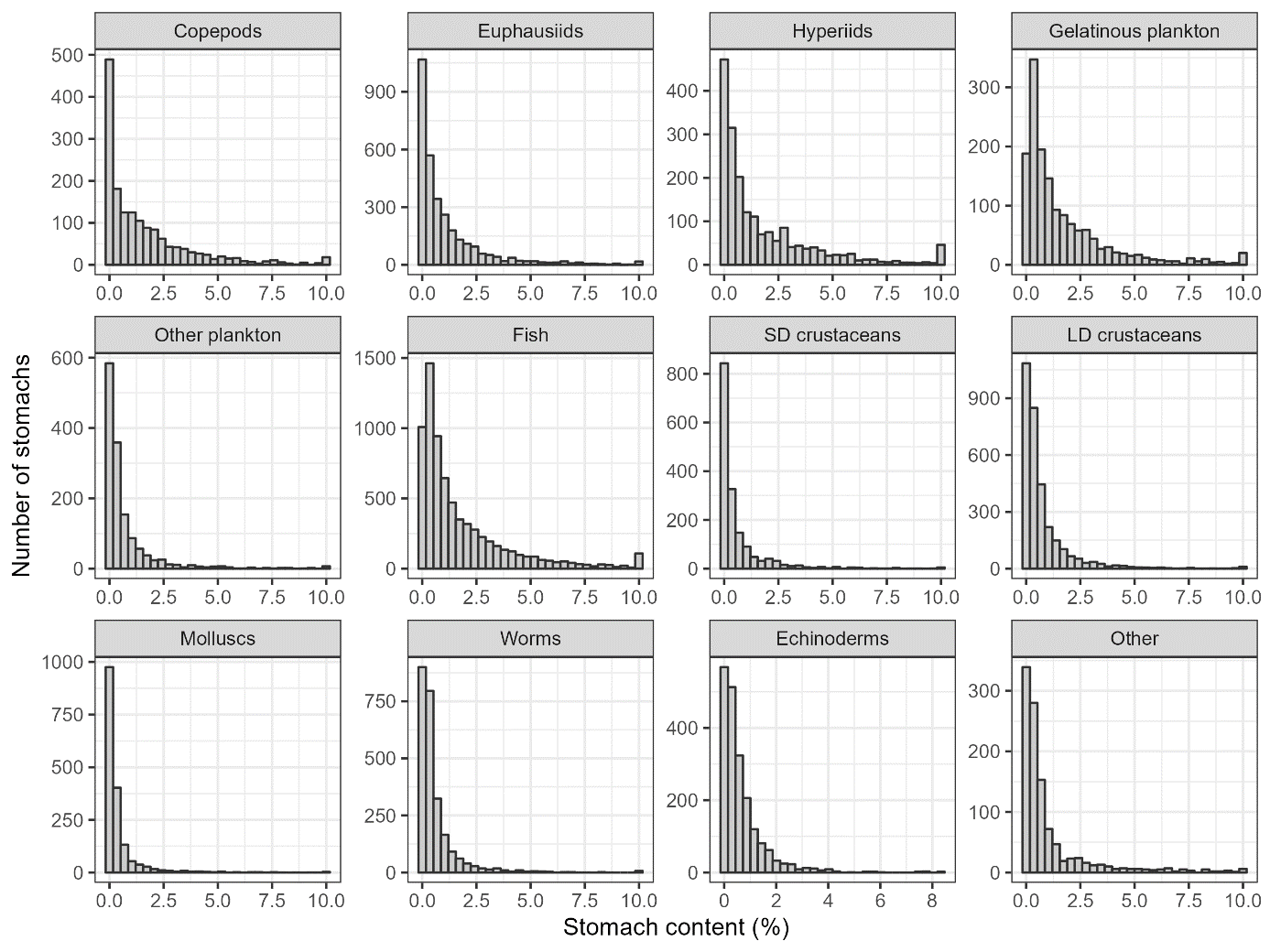
**

Fig. S-1. Frequency distributions of number of stomachs versus stomach content (weight as % weight of fish predator) for 12 prey categories. The last bin at 10 % includes all stomachs with >10 % content (see Table 2).


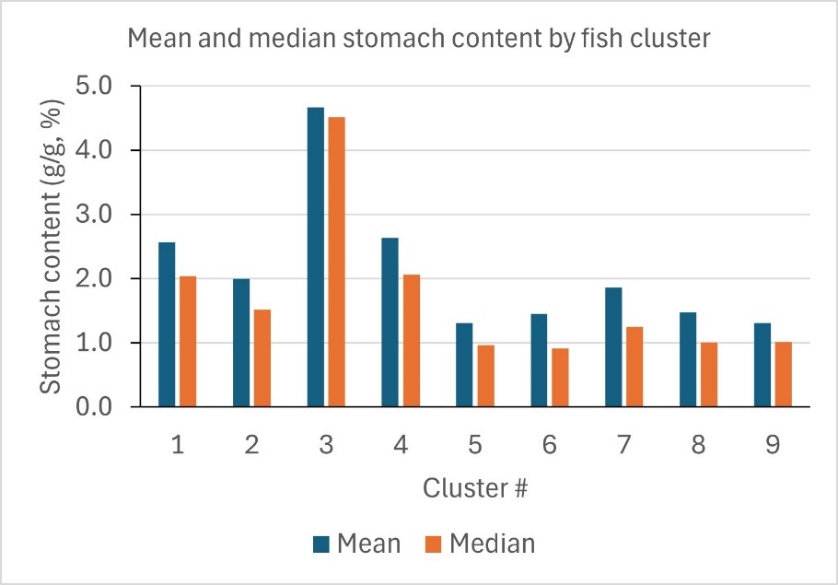


Fig. S-2. Mean (arithmetic) and median stomach content (g weight per weight of fish, expressed as %) for groups (clusters) of fish species from Eriksen et al. (2020). See Table 3 for list of species.

Figure S-3 on this and following four pages.


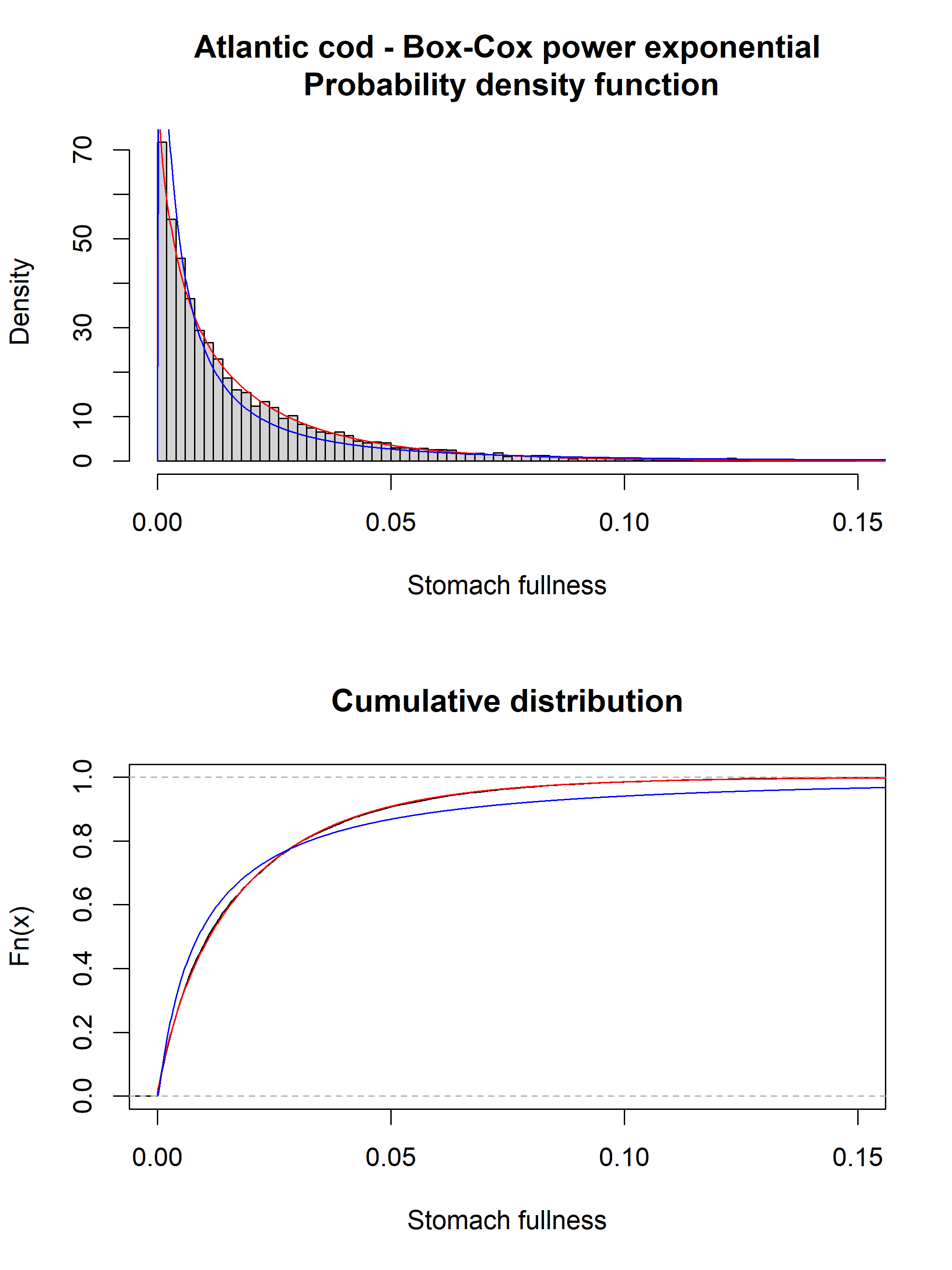

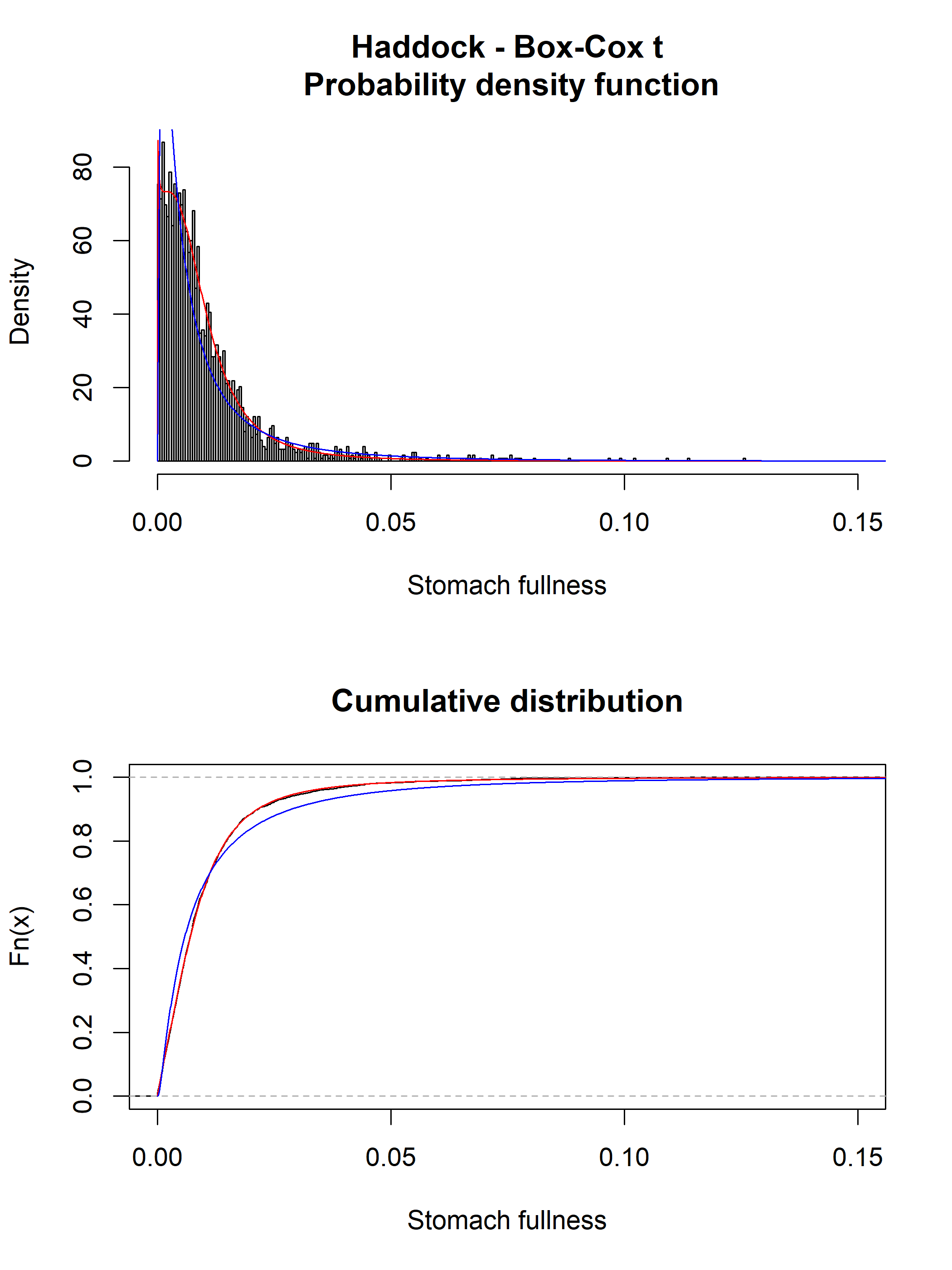


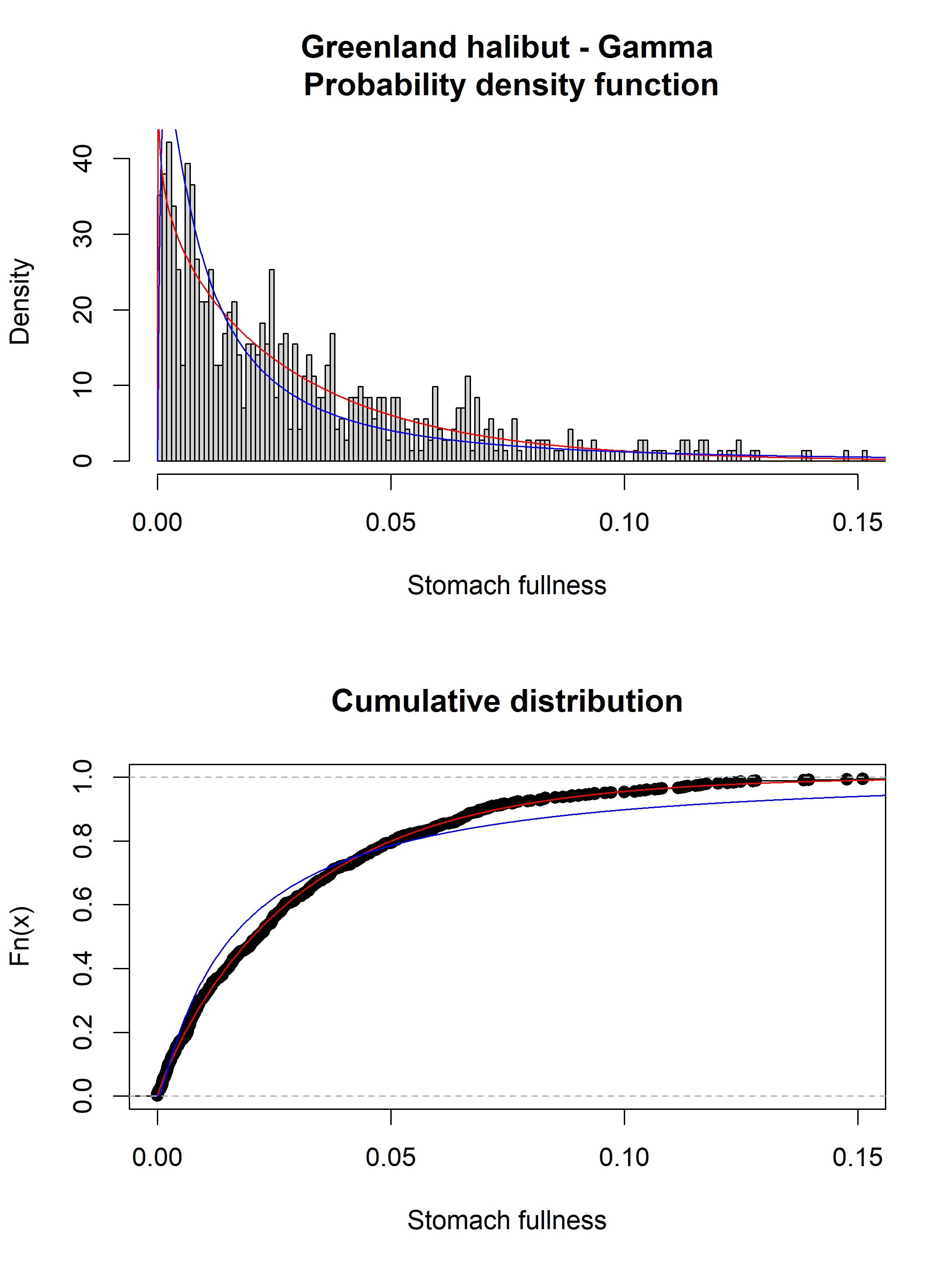

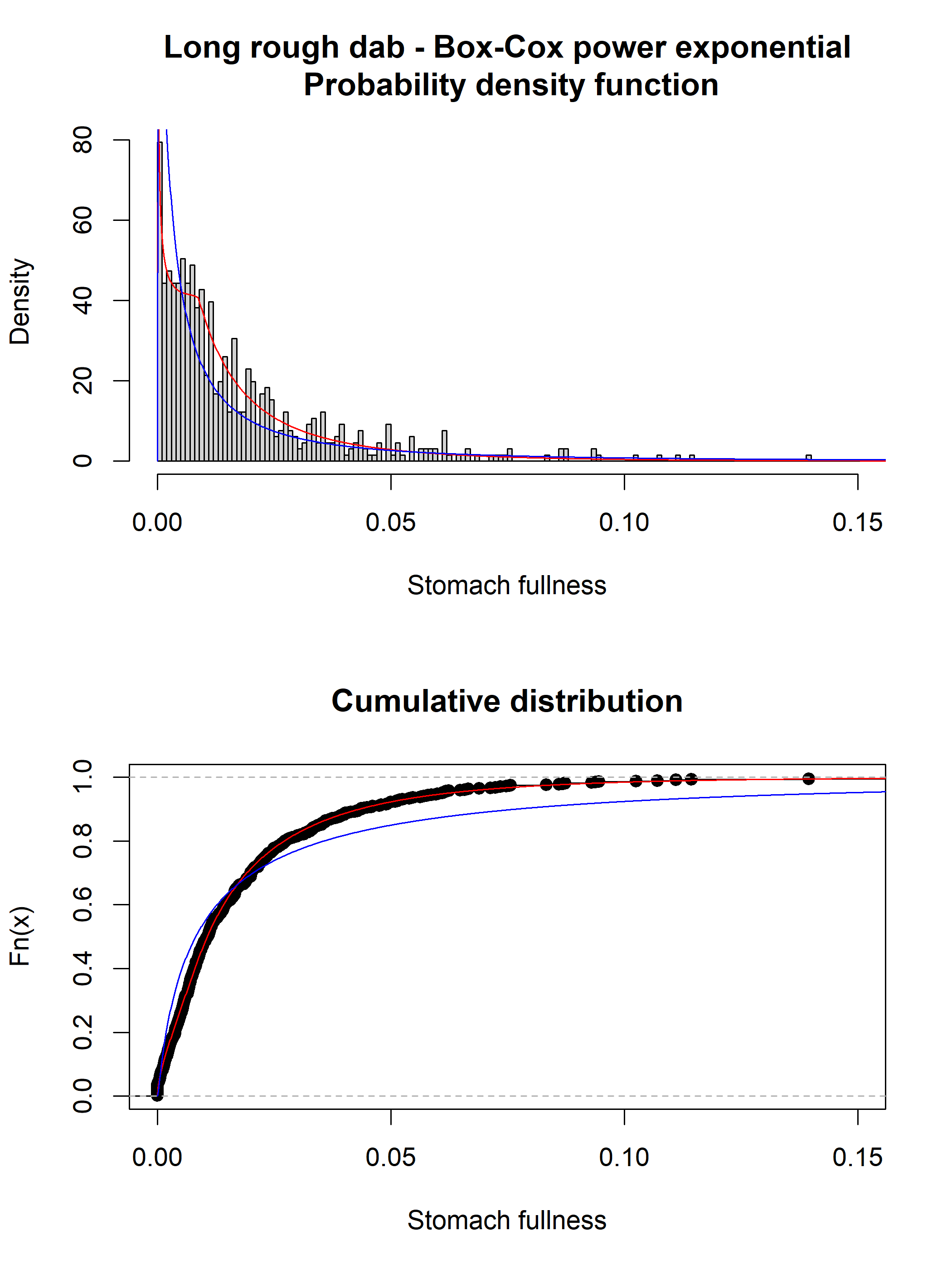


Fig. S-3. Species – Atlantic cod (upper left), haddock (upper right), Greenland halibut (lower left), long rough dab (lower right).


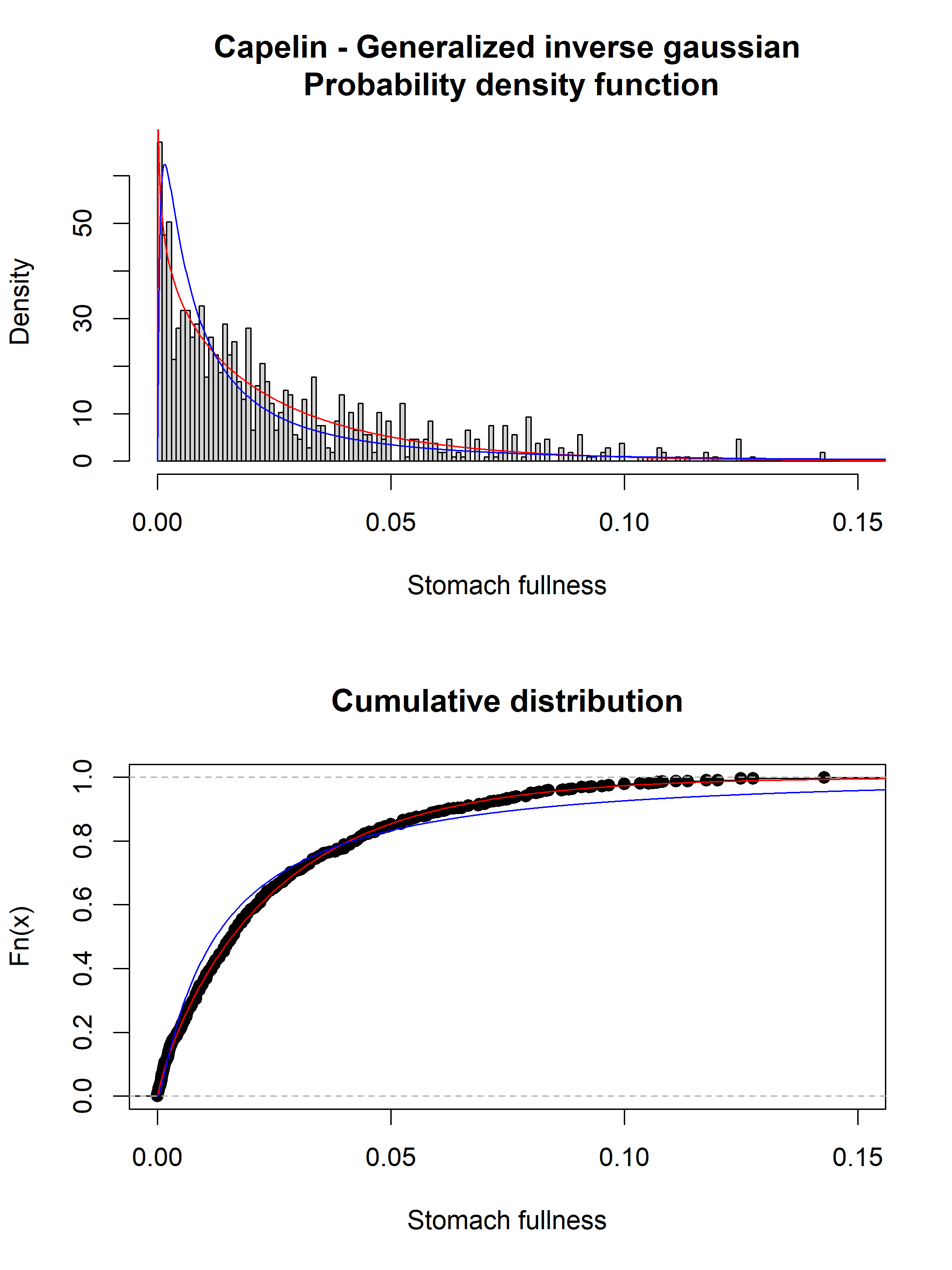

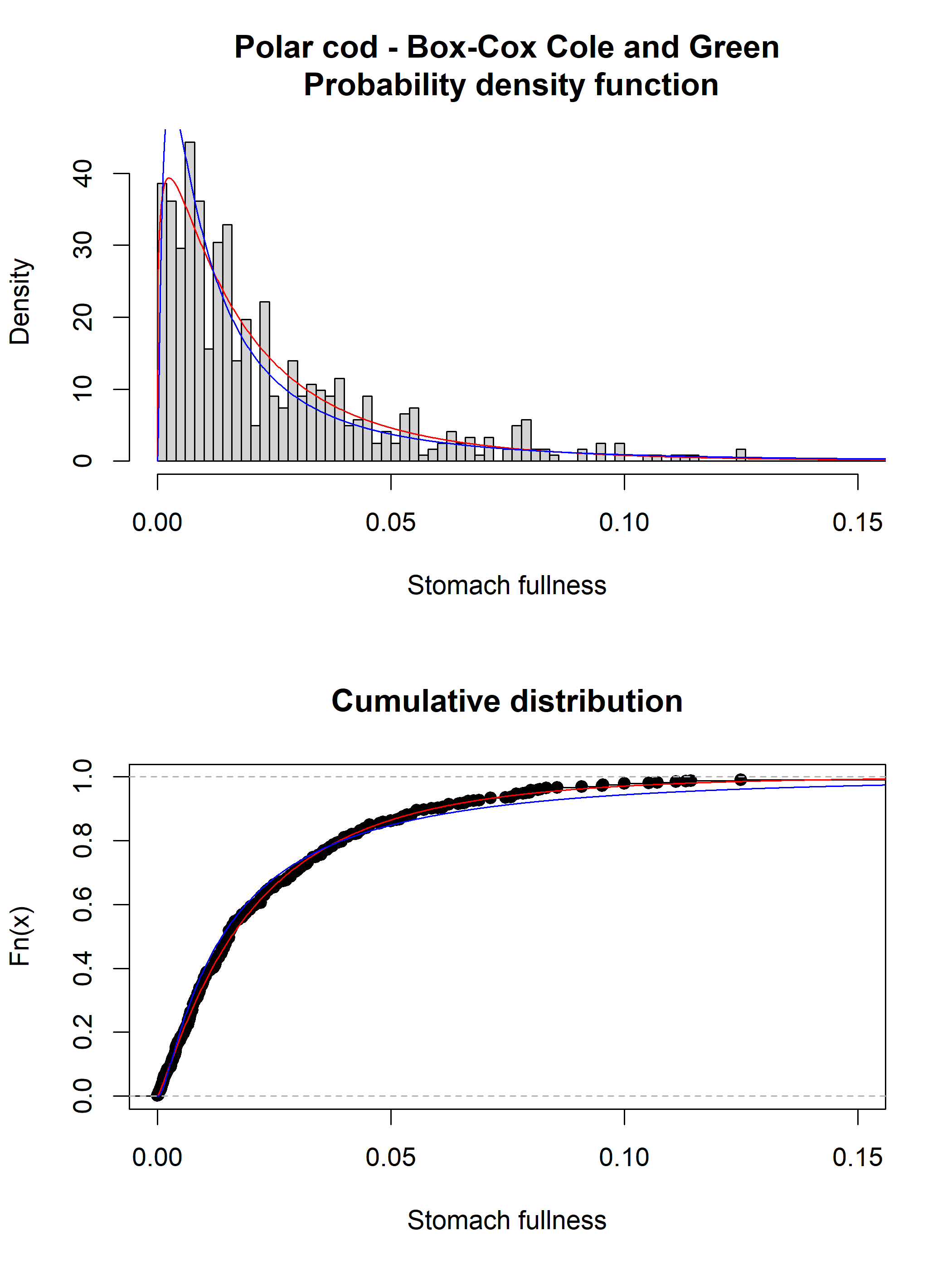


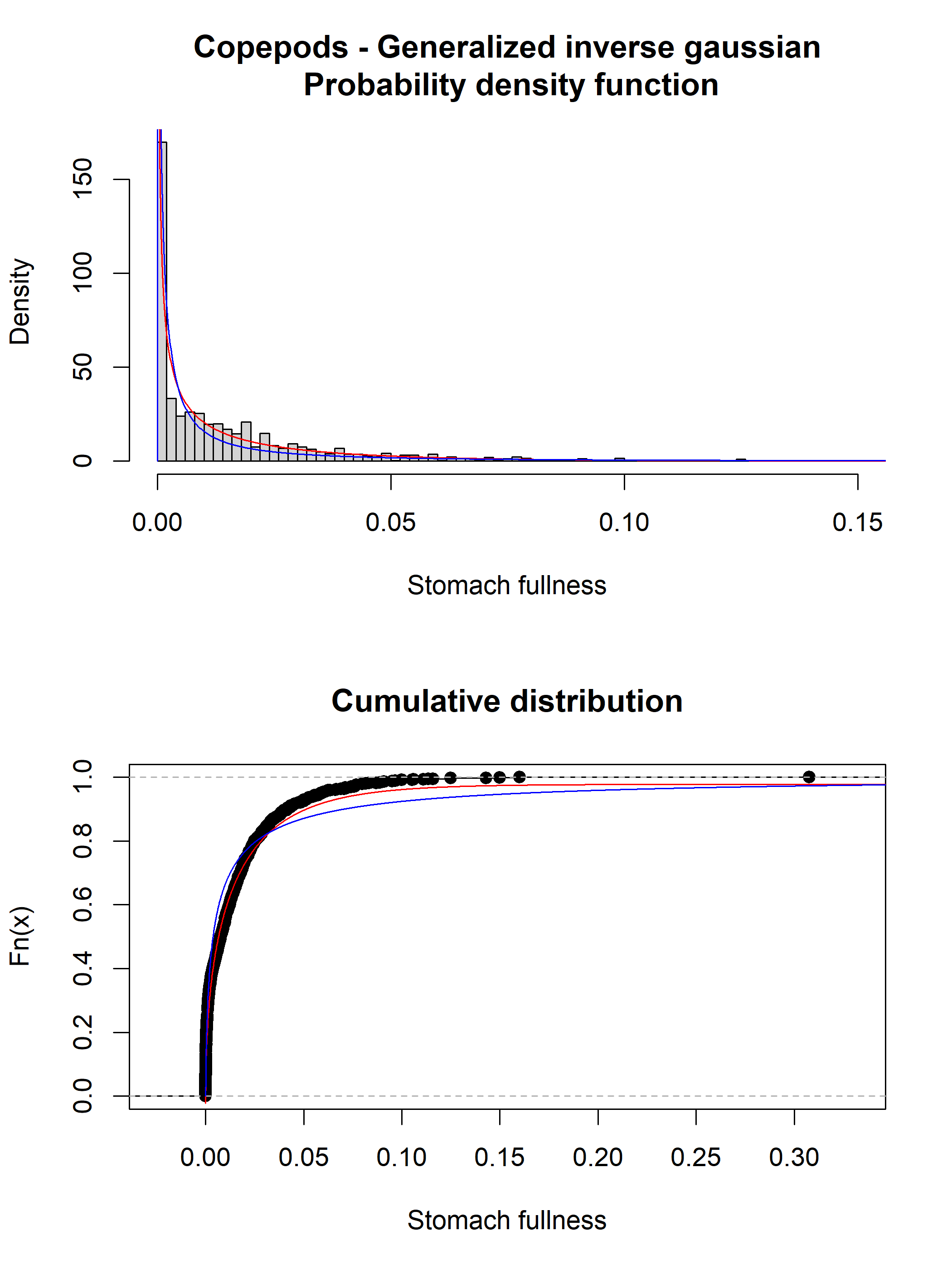

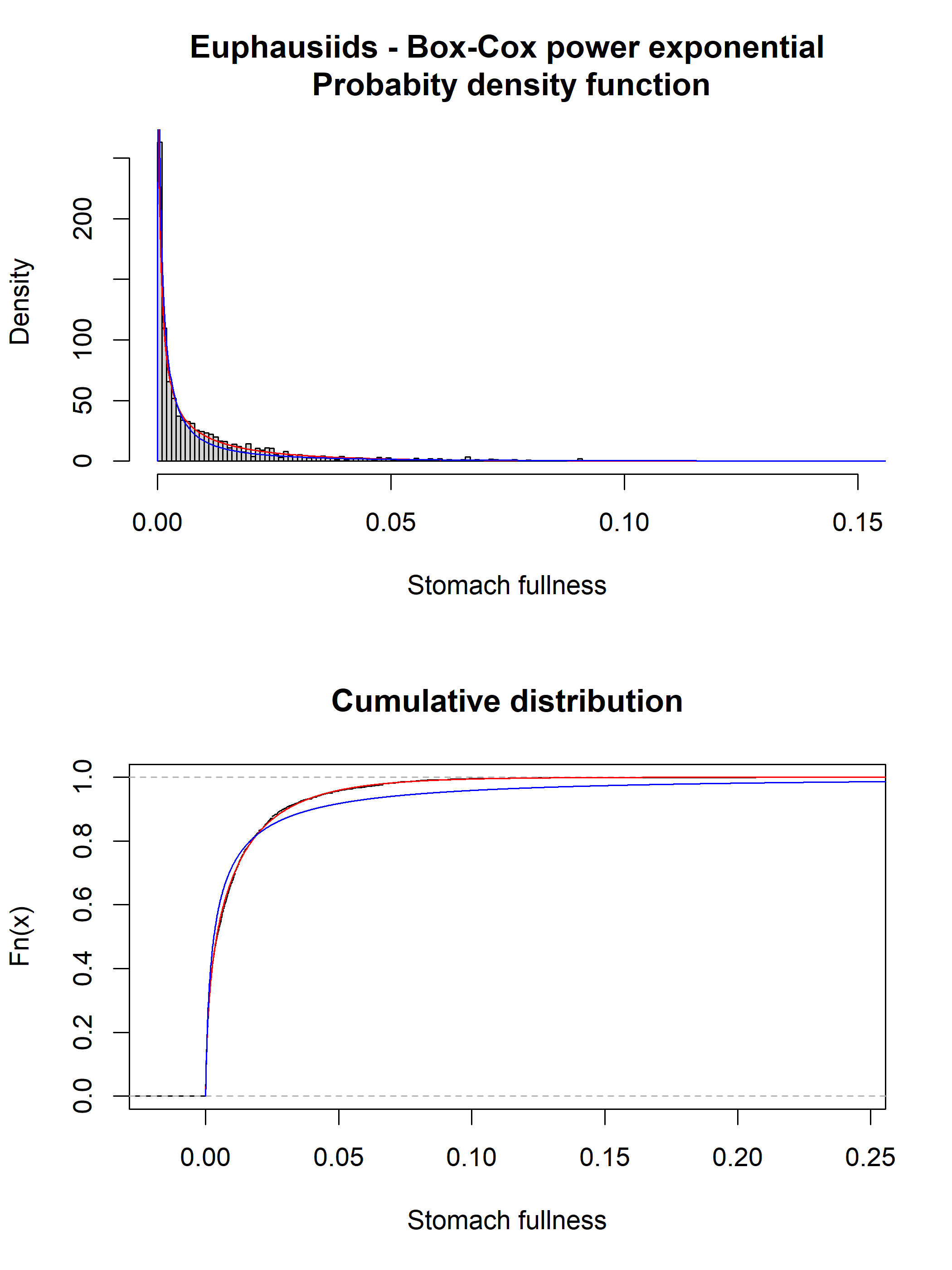


Fig. S-3. Species – capelin (upper left), polar cod (upper right). Prey types – copepods (lower left), euphausiids (lower right).


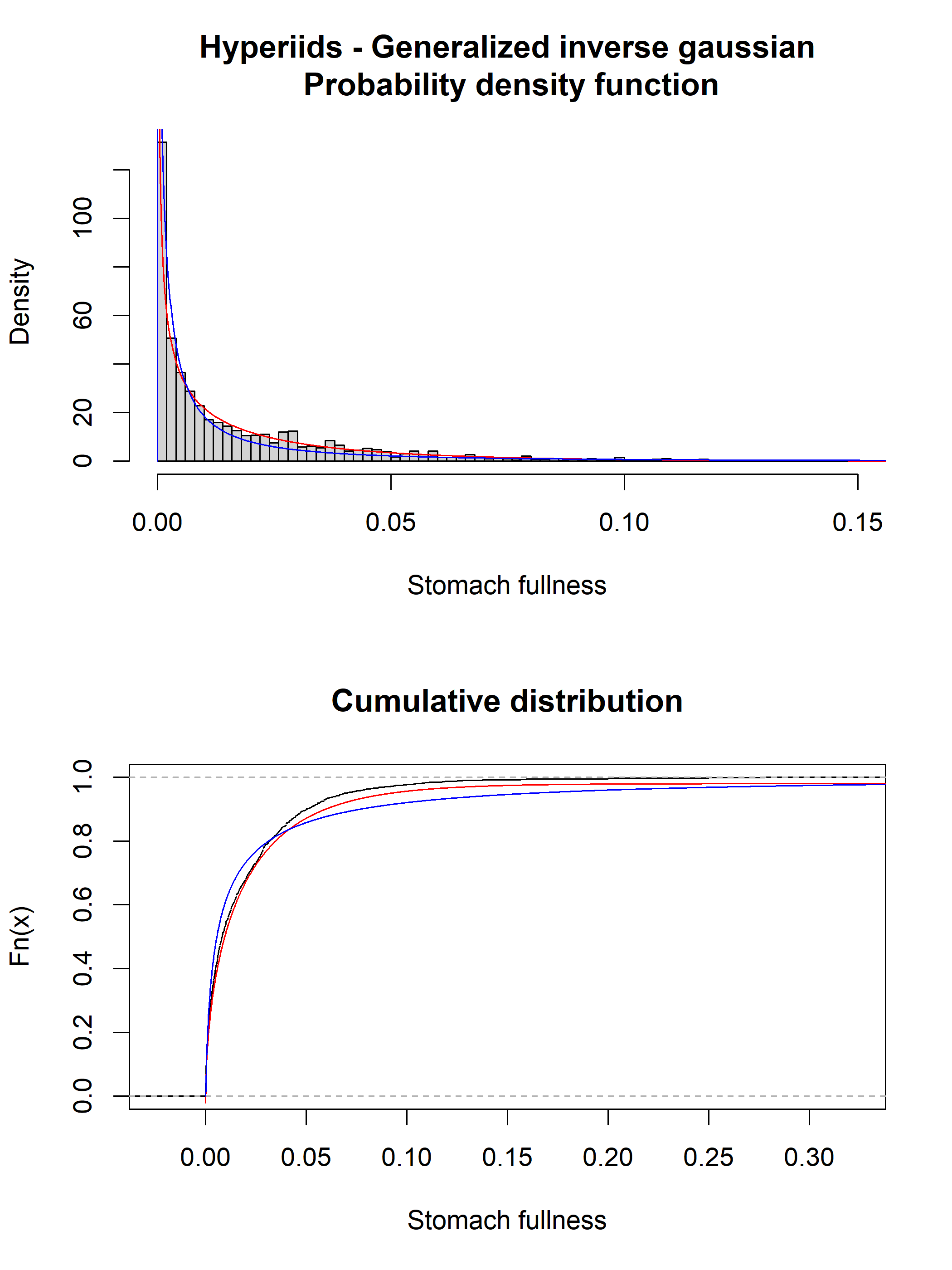

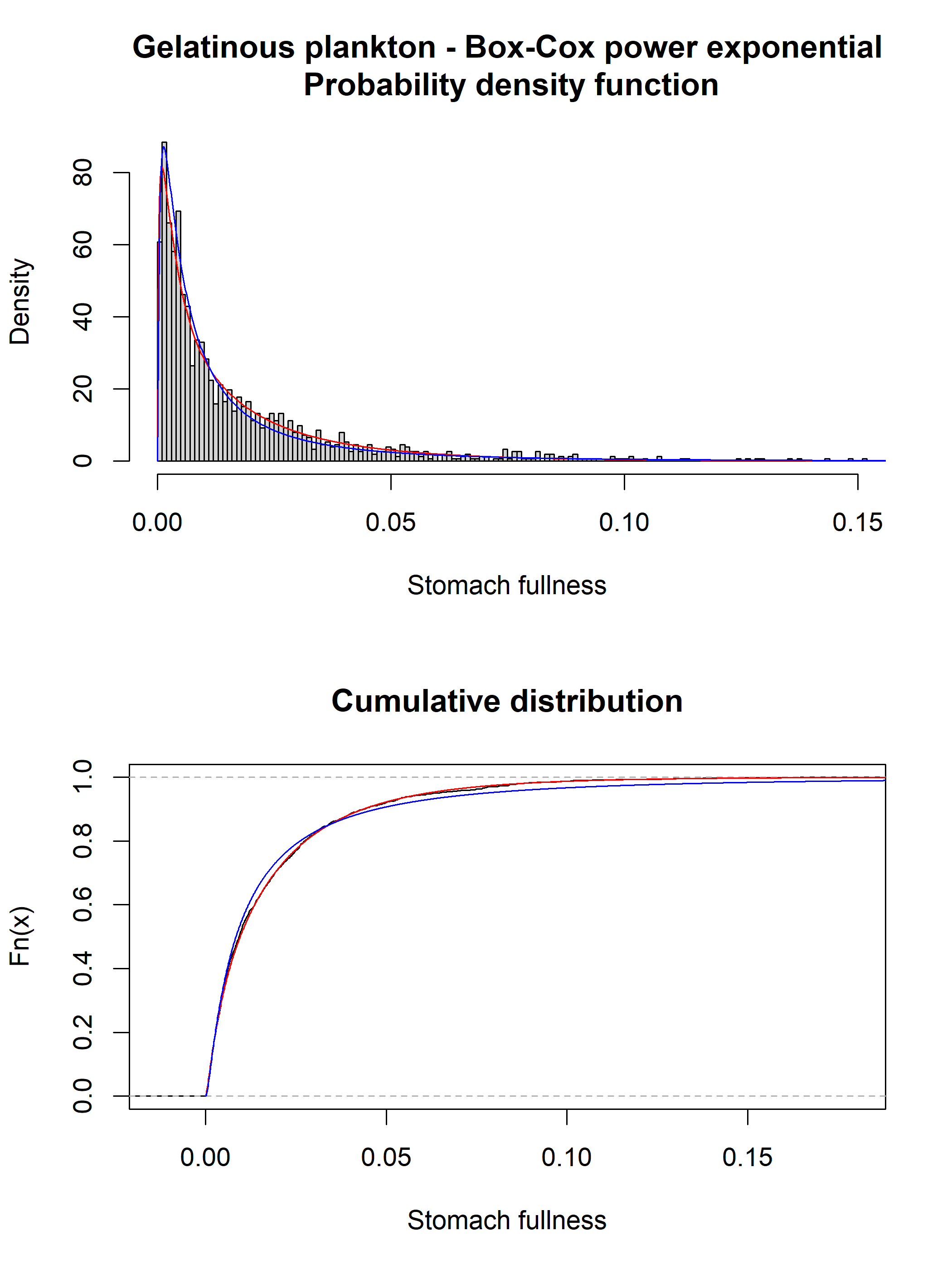


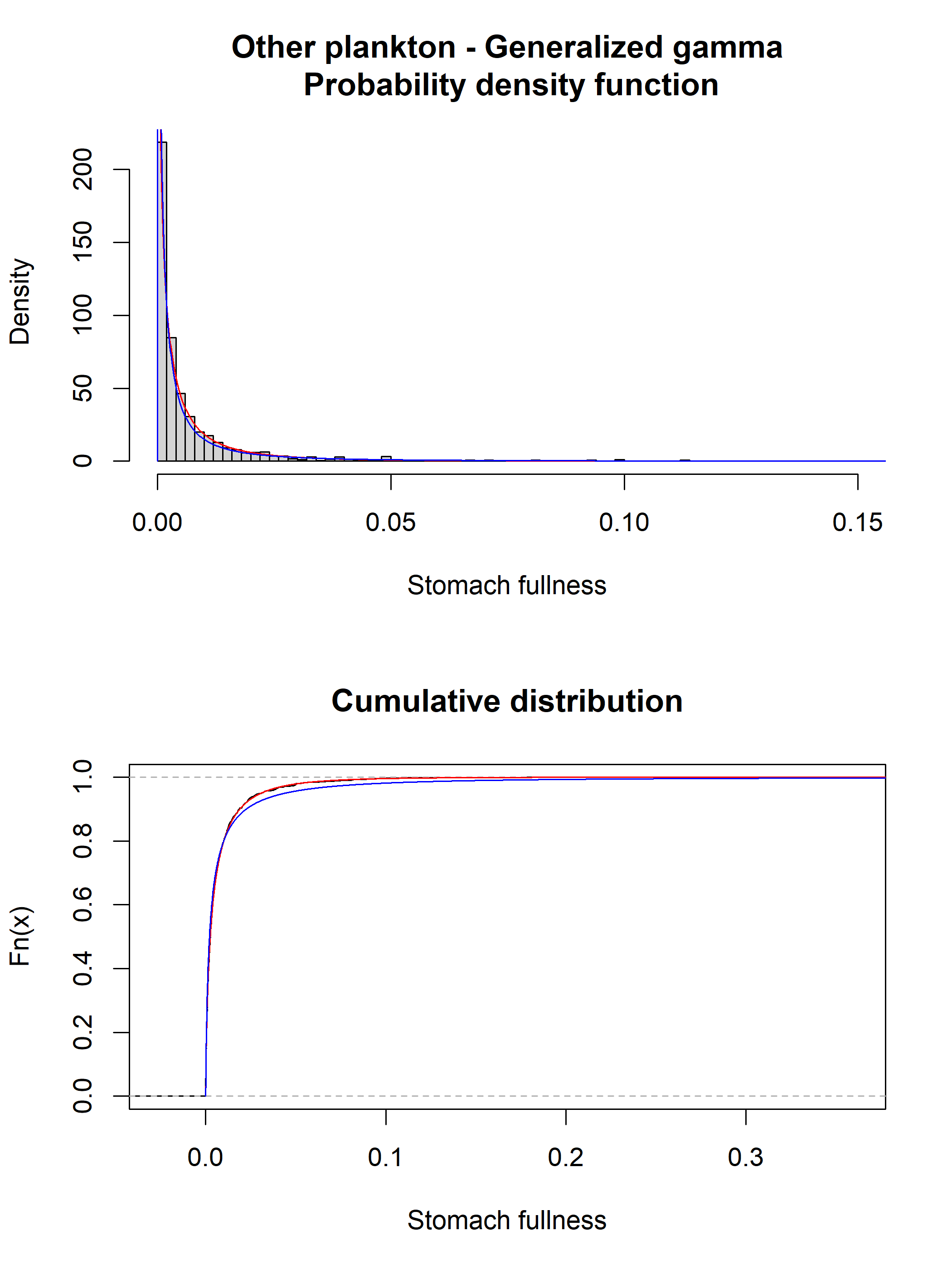

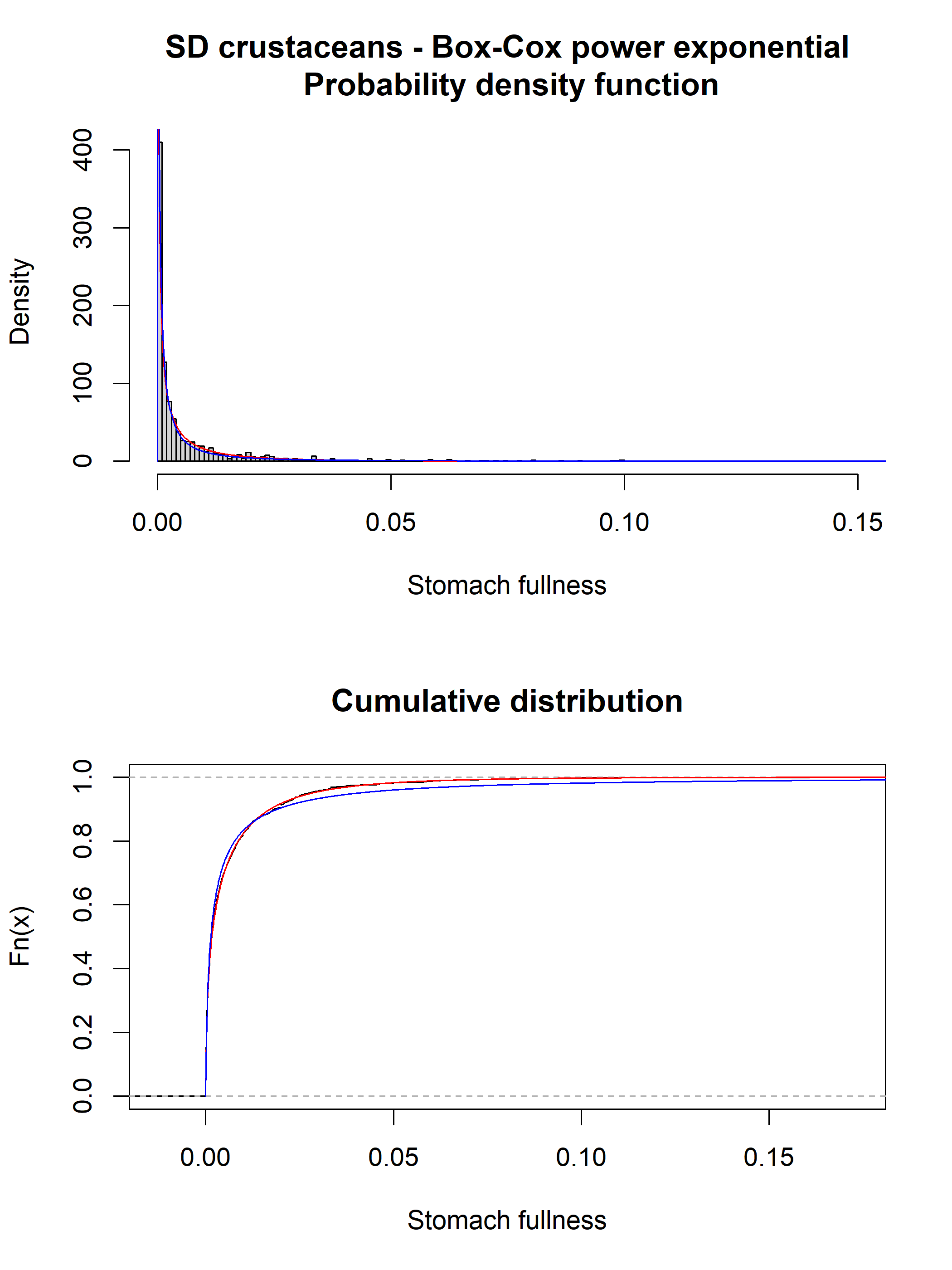


Figure S-3. Prey types – Hyperiids (upper left), gelatinous plankton (upper right), other plankton (lower left), small demersal (SD) crustaceans (lower right).


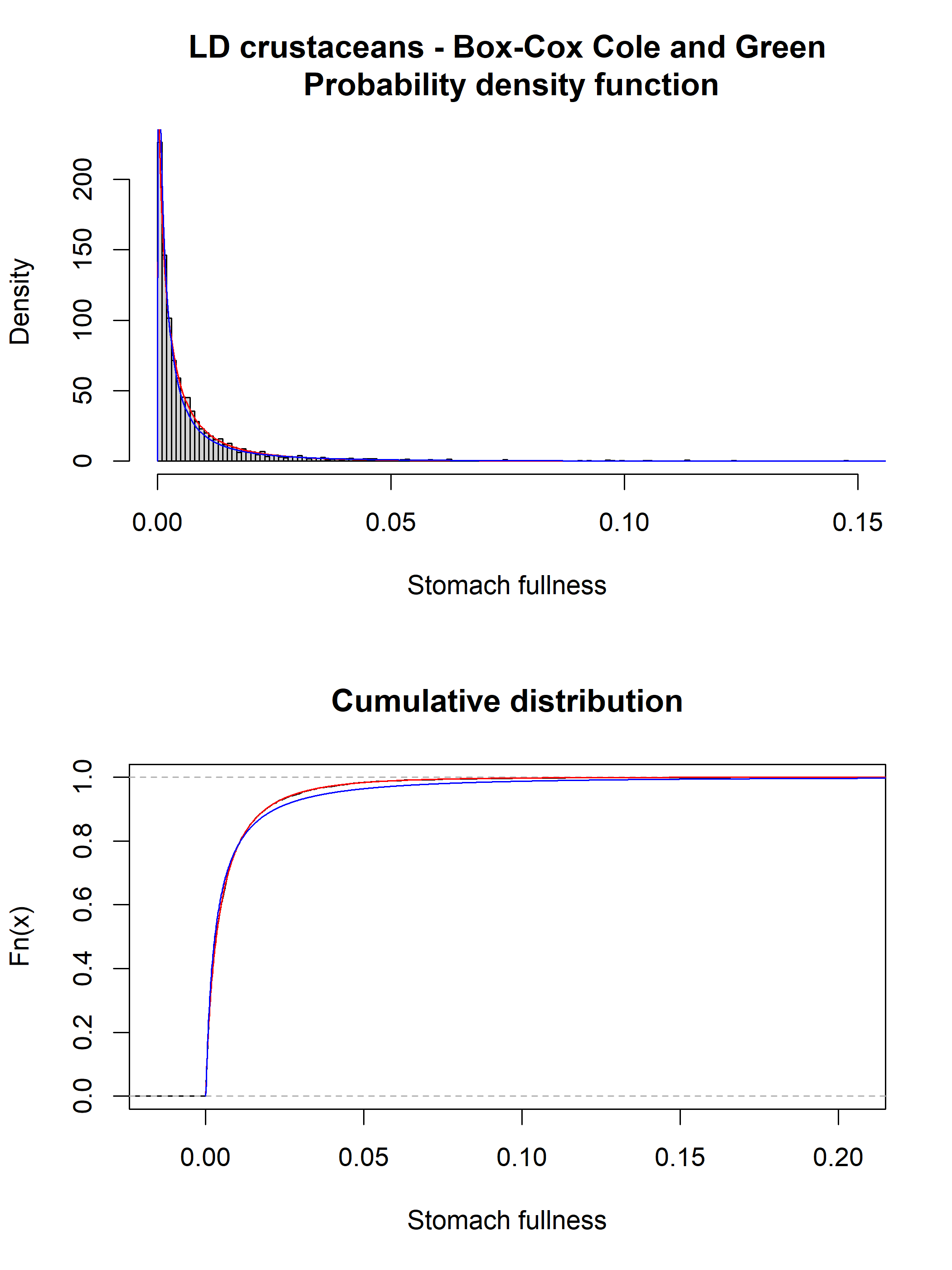

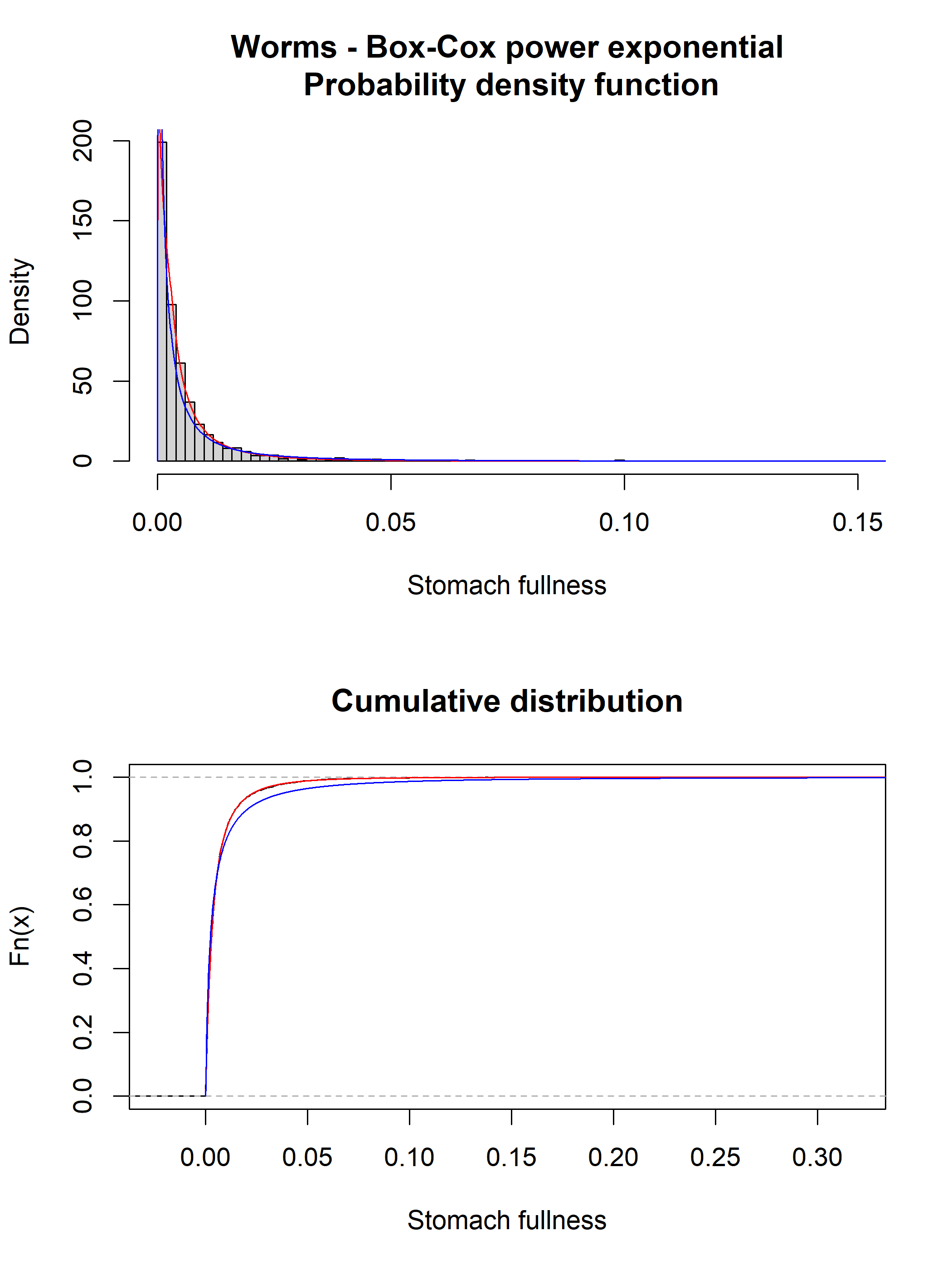


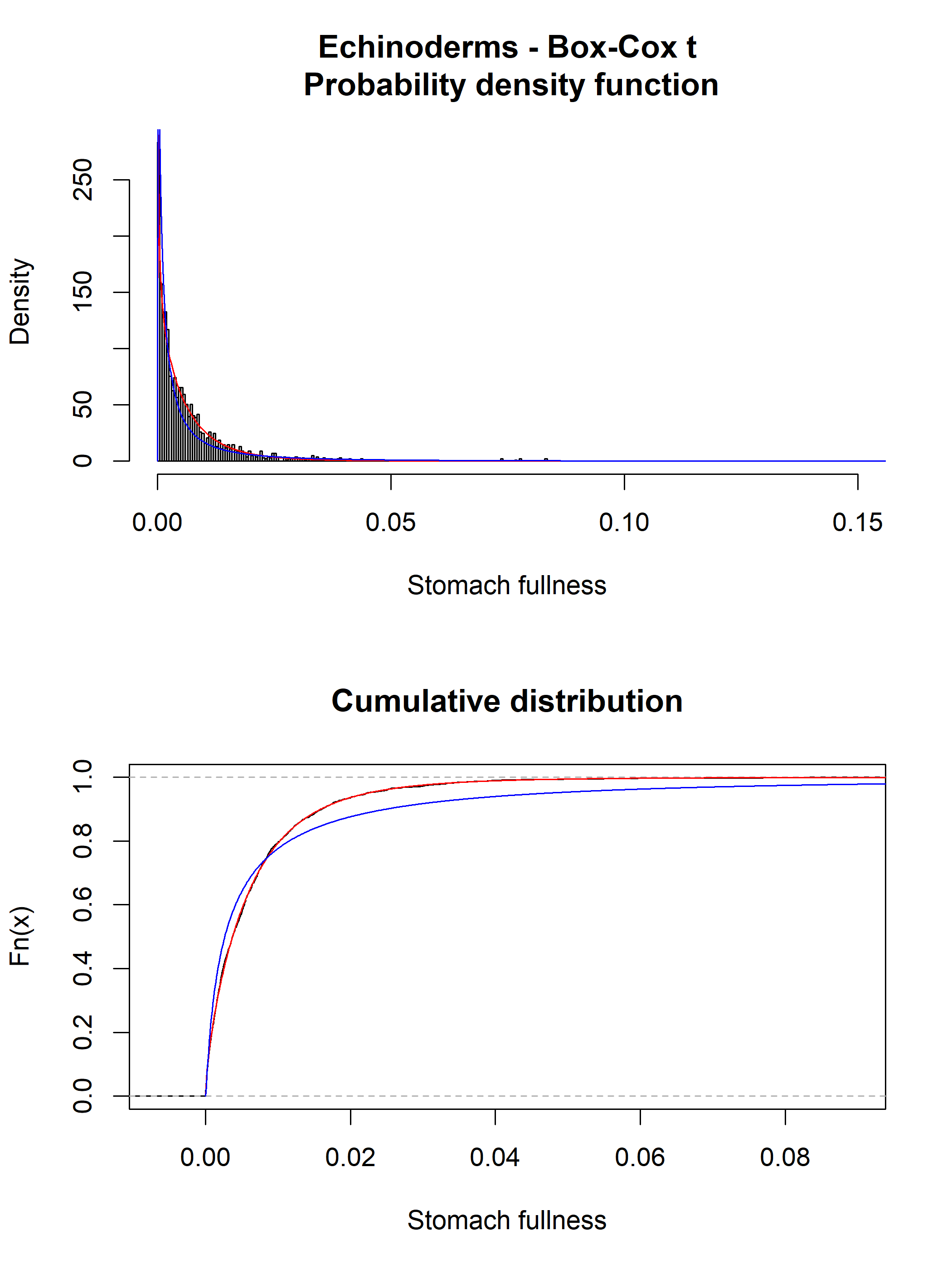

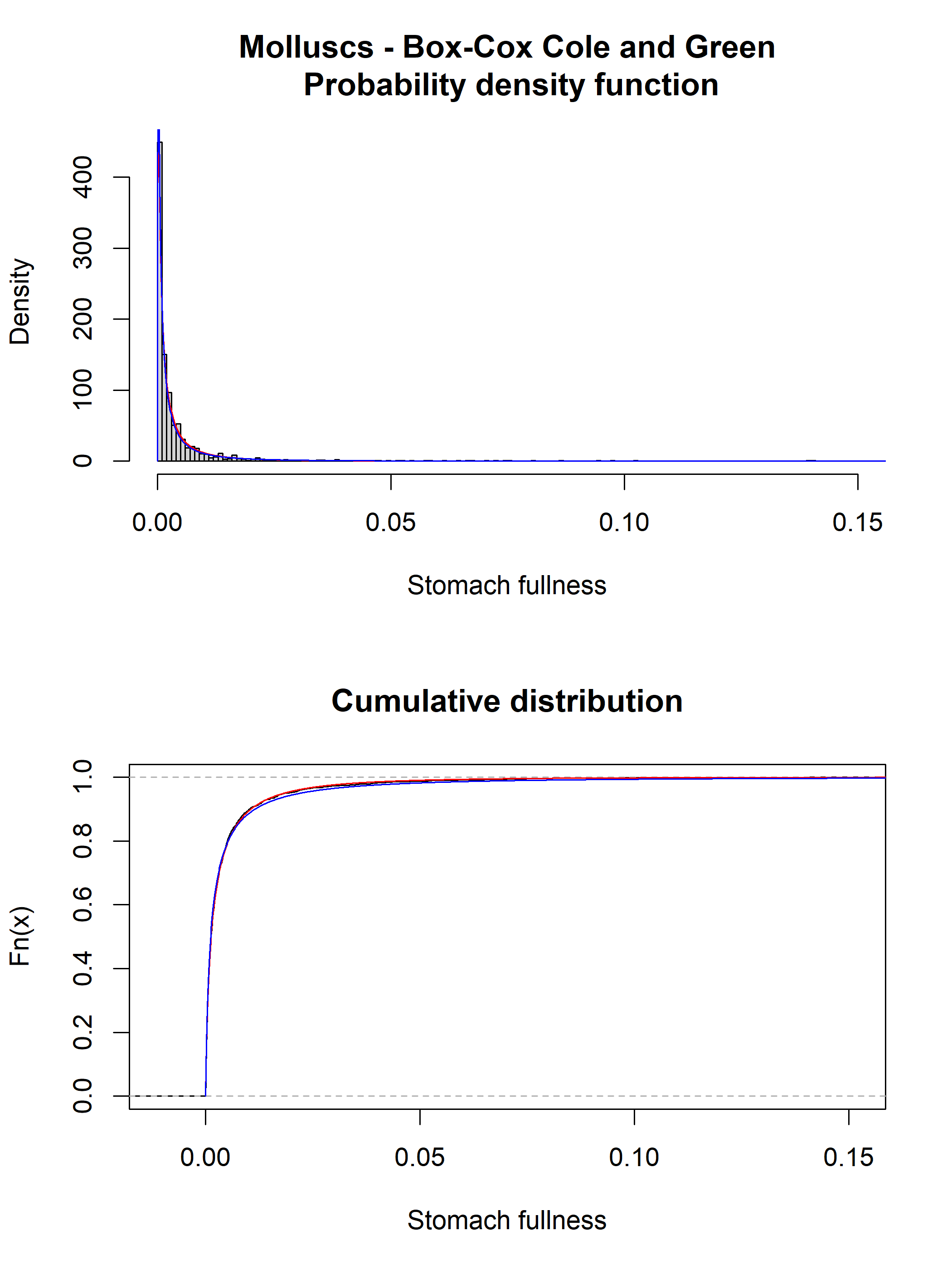


Fig. S-3. Prey types – Large demersal (LD) crustaceans (upper left), worms (upper right), echinoderms (lower left), molluscs (lower right).


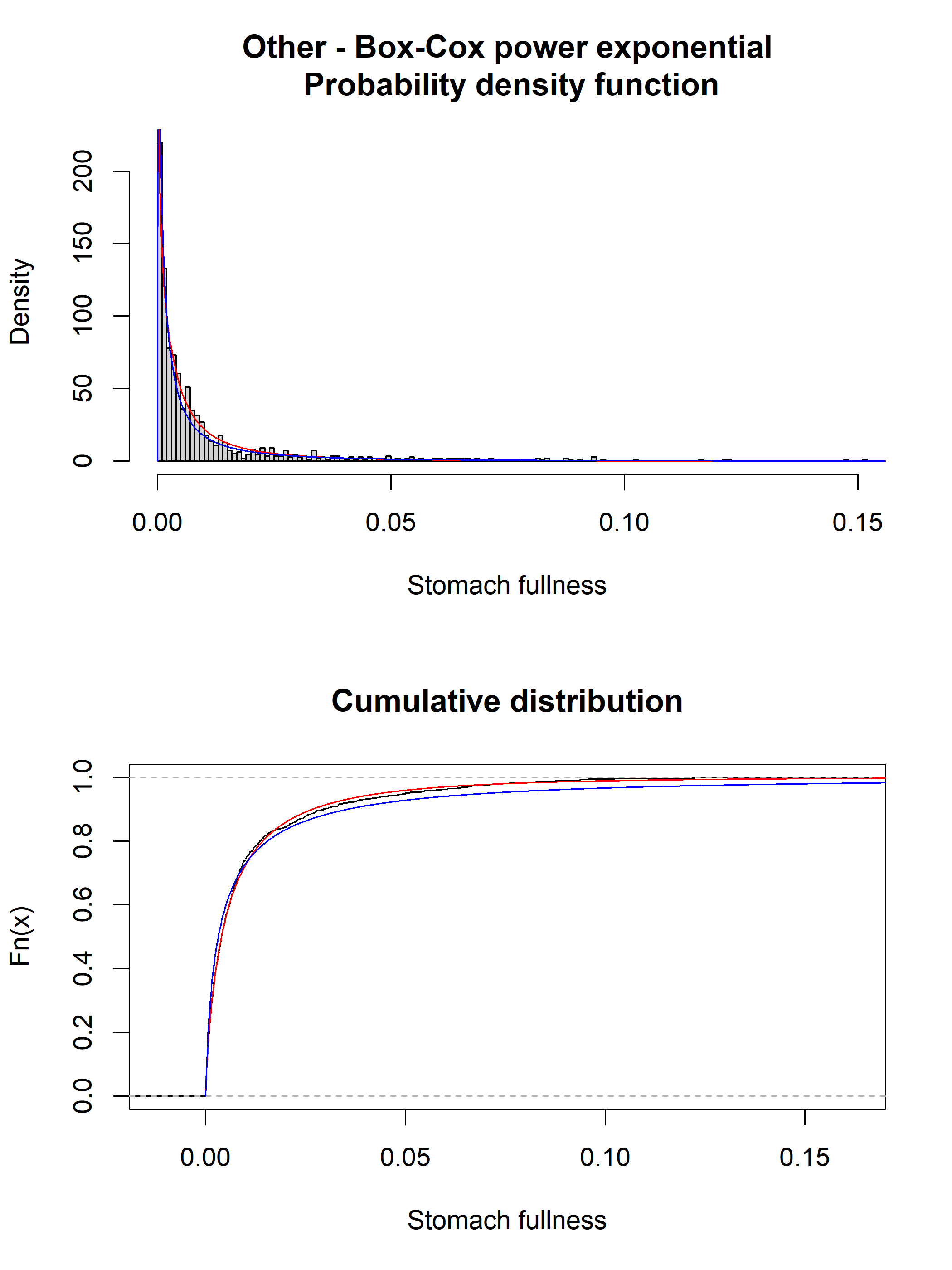

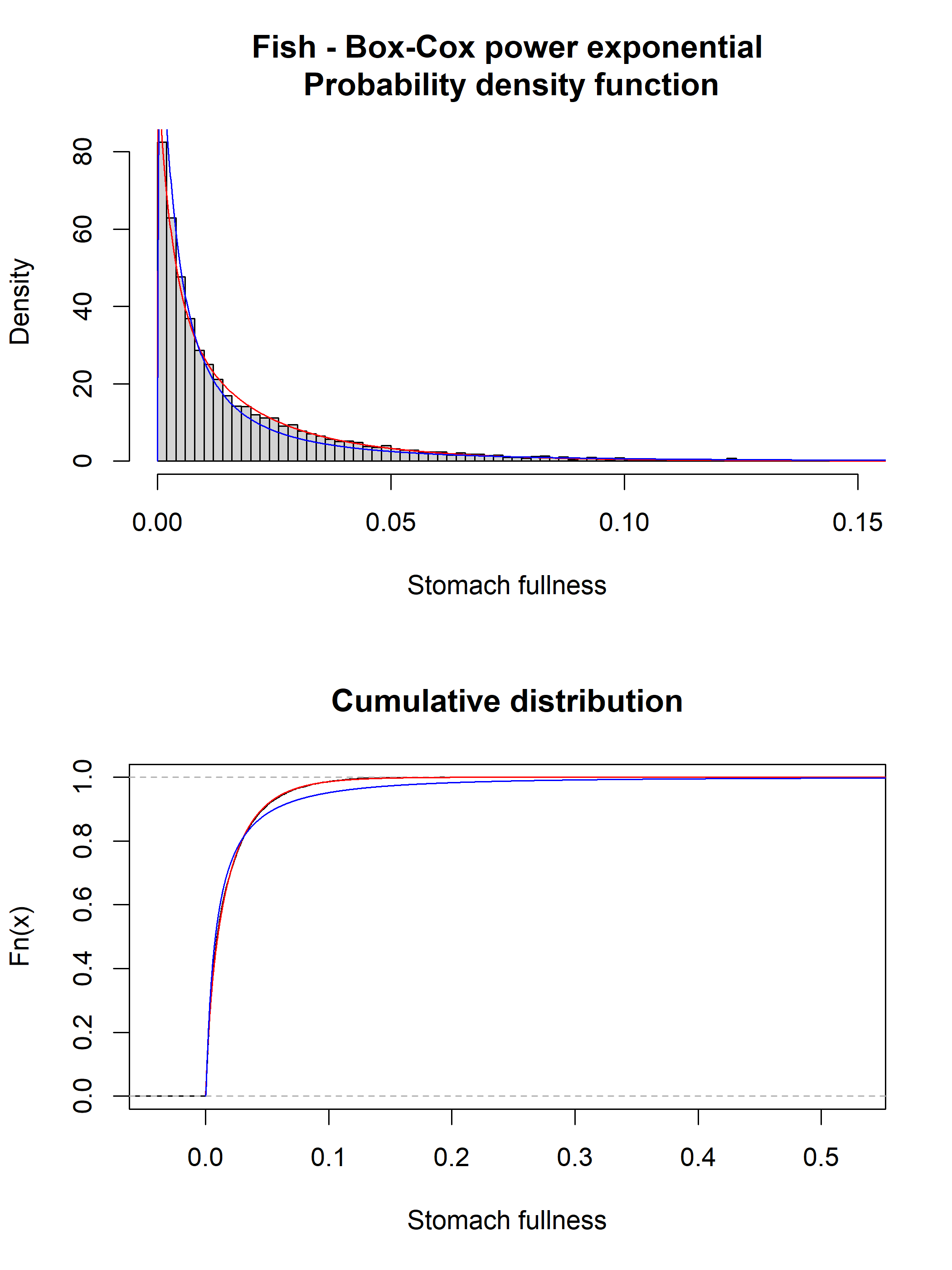


Fig. S-3. Prey types – Other prey (left), fish (right).

Fig. S-3. Frequency distribution (histogram) and probability density function (pdf) of total stomach content for six species of fish predators, and of stomach content of 12 different types of prey across all predator fish species. The six species of fish are: Atlantic cod, haddock, Greenland halibut, long rough dab, capelin, and polar cod. The 12 types of prey are (see Table 1): copepods, euphausiids, hyperiid amphipods, gelatinous zooplankton, other plankton, small demersal (SD) crustaceans, large demersal (LD) crustaceans, worms, echinoderms, molluscs, other prey, and fish. The model with best fit to the data is shown as a red line and is identified in the figure heading of each case (see Table 4). For comparison, the log-normal distribution is shown as a blue line. Two panels are shown in each of the 18 cases (6 species and 12 prey types) – the upper is frequency distribution and pdf with data on linear scale, and the lower is the cumulative version. Note that the cumulative frequency distribution shown as a black line is overlaid with near perfect match by the red line for the best model and is not visible in most cases. Stomach fulness on the x-axis is the stomach content relative to the weight of the fish (g g^-1^).


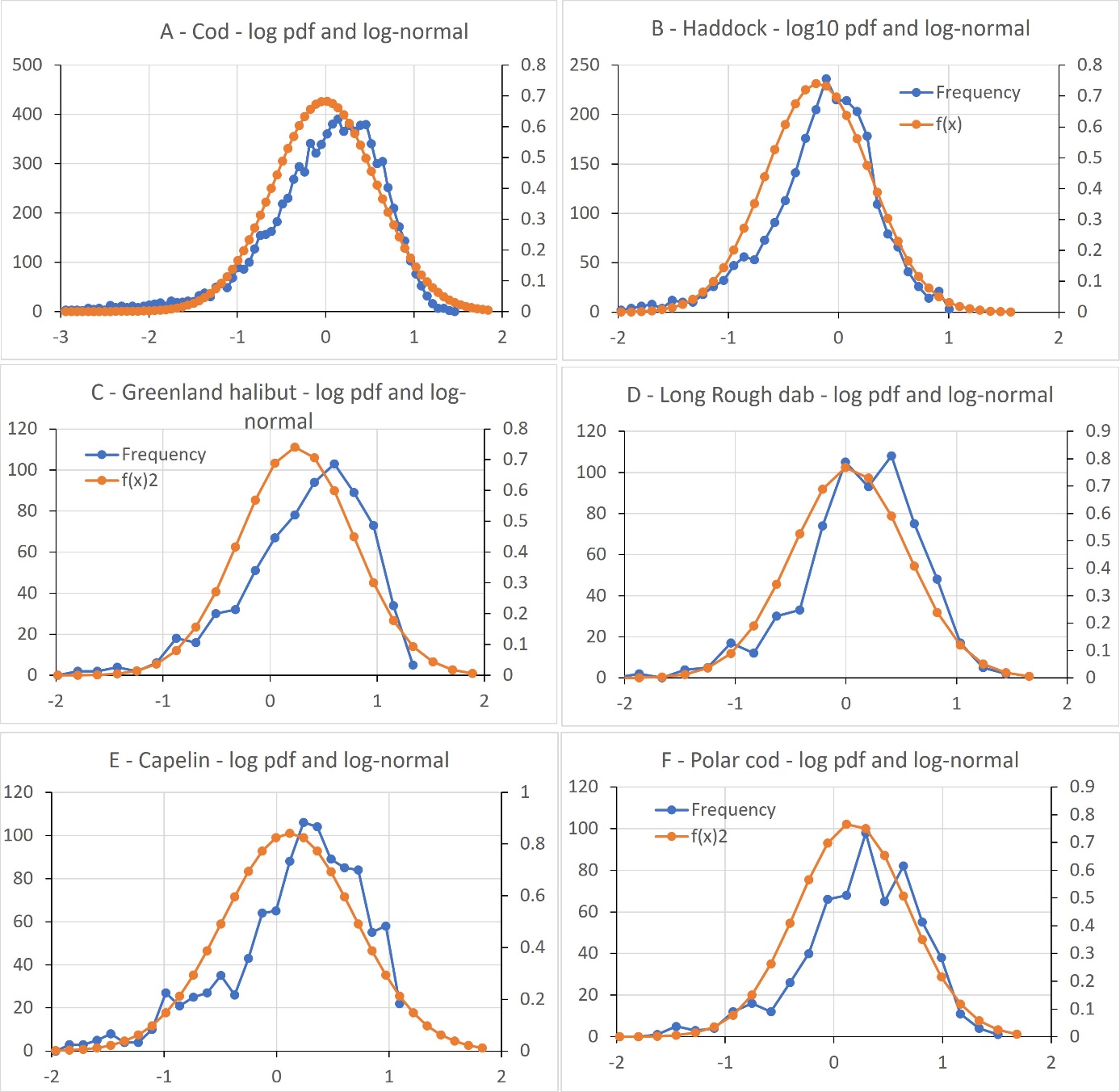


Fig. S-4. Frequency distributions (blue; number of observations on y-axis to the left) for weight of total stomach content (log10-transformed %-weight, x-axis) for six species of fish predators: Atlantic cod (A), haddock (B), Greenland halibut (C), long rough dab (D), capelin (E), and polar cod (F). Log-normal distributions (orange) are included for comparison, calculated from values of mean and standard deviation for each of the species (y-axis to the right).


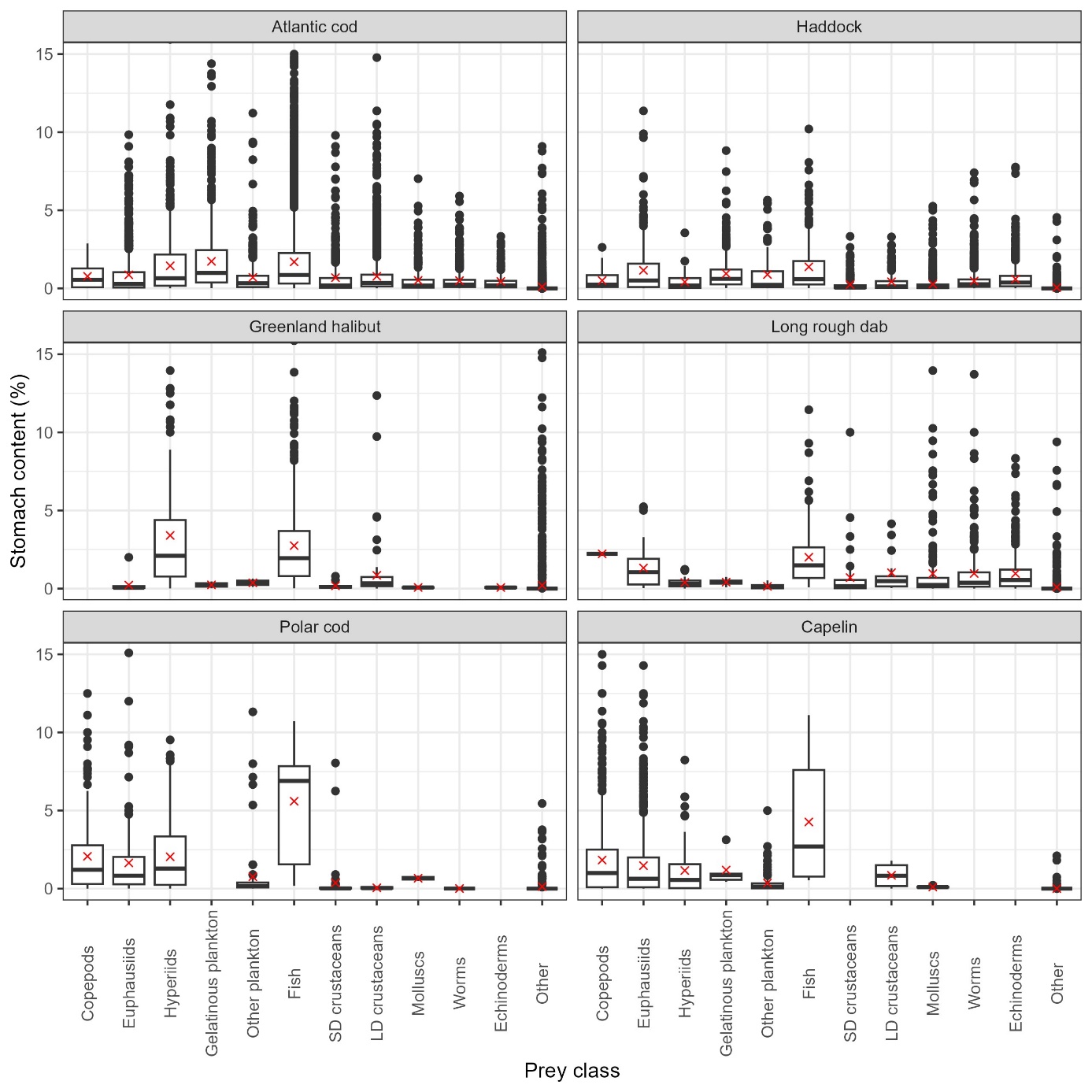


Fig. S-5. Box-whisker plots of stomach contents (%-weight) of 12 prey categories (see Table 1) for six species of fish predators: Atlantic cod, haddock, Greenland halibut, long rough dab, polar cod, and capelin. Figures show median (horizontal bar), mean (red cross), 25-75 percentile (box), 5-95 percentile (vertical line), and statistical outliers (points).


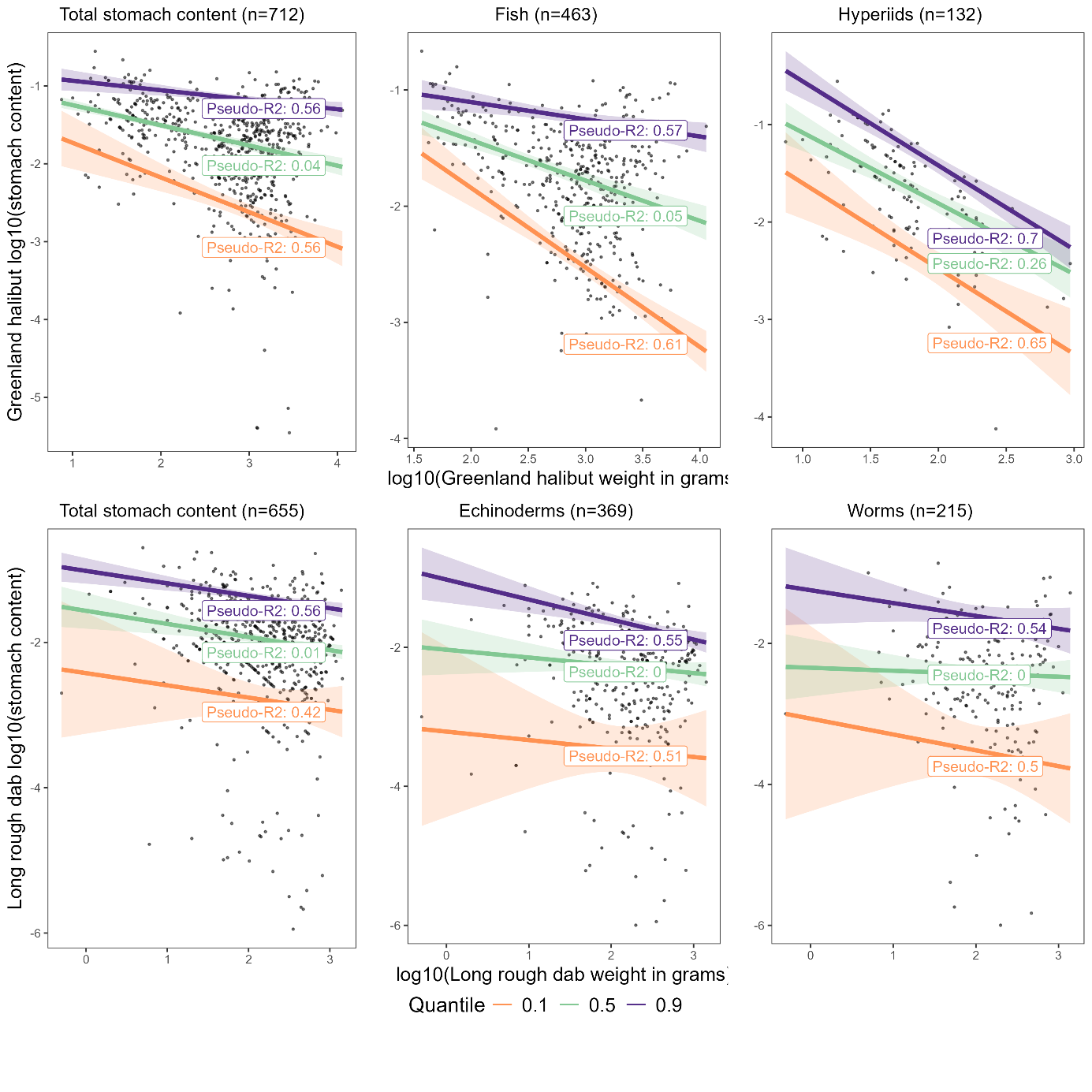
Fig. S-6. Stomach content (weight of prey per weight of predator fish) versus individual size (weight) of fish predators for Greenland halibut (upper row) and long rough dab (lower row). The three panels for each species show total stomach content, and contents of fish and hyperiid amphipod prey for Greenland halibut, and contents of echinoderm and worm prey for long rough dab. The data are log10 transformed. The upper and lower violet and orange lines are quantile regression lines for upper and lower (90 % and 10 %) quantiles, while the middle green line is the overall (median) regression. The shaded bands are 95 % confidence bands around the regression lines.


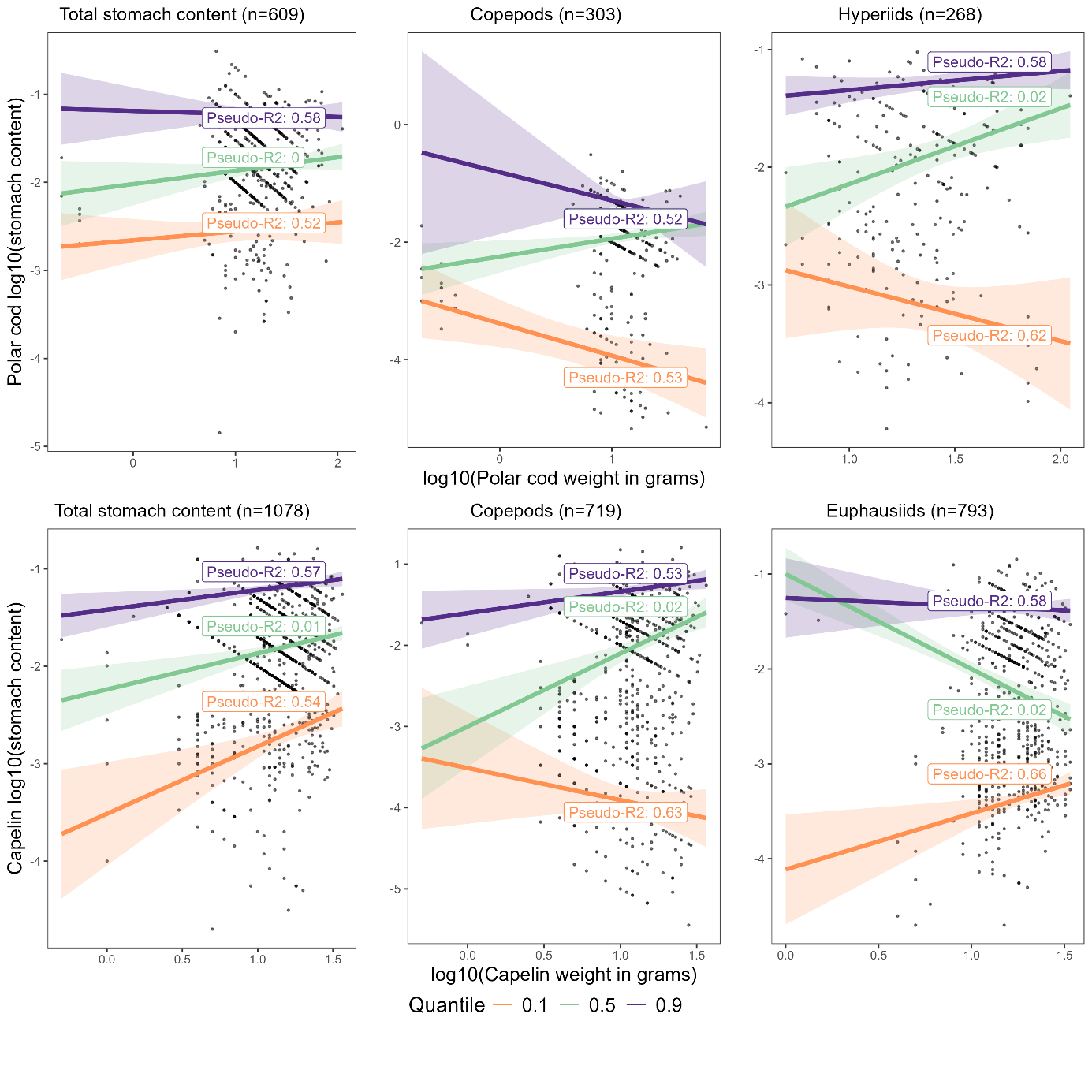


Fig. S-7. Stomach content (weight of prey per weight of predator fish) versus individual size (weight) of fish predators for polar cod (upper row) and capelin (lower row). The three panels for each species show total stomach content, and contents of copepod and hyperiid amphipod prey for polar cod, and contents of copepod and euphausiid prey for capelin. The data are log10 transformed. The upper and lower violet and orange lines are quantile regression lines for upper and lower (90 % and 10 %) quantiles, while the middle green line is the overall (median) regression. The shaded bands are 95 % confidence bands around the regression lines.
